# Epigenetically regulated lncRNAs dissect the intratumoural heterogeneity and facilitate immune evasion of glioblastomas

**DOI:** 10.7150/thno.79874

**Published:** 2023-03-05

**Authors:** Dahua Xu, Meng Cao, Bo Wang, Xiaoman Bi, Haiying Zhang, Deng Wu, Chunrui Zhang, Jiankai Xu, Zhizhou Xu, Dehua Zheng, Liyang Chen, Peihu Li, Hong Wang, Yan Liu, Hongyan Jiang, Kongning Li

**Affiliations:** 1 Hainan General Hospital, Hainan Affiliated Hospital of Hainan Medical University, Haikou, 570311, China.; 2 Key Laboratory of Tropical Translational Medicine of Ministry of Education, College of Biomedical Information and Engineering, Hainan Medical University, Haikou, 571199, China; 3 Key Laboratory of Tropical Translational Medicine of Ministry of Education, College of Pharmaceutical, Hainan Medical University, Haikou, 571199, China; 4 School of Life Sciences, Faculty of Science, The Chinese University of Hong Kong, 999077, Hong Kong; 5 Institute of Genetics and Developmental Biology, Chinese Academy of Sciences, Beijing 100020, China; 6 College of Bioinformatics Science and Technology, Harbin Medical University, Harbin 150081, China

**Keywords:** glioblastomas, DNA methylation, long non-coding RNAs, cancer subtypes, immune microenvironment

## Abstract

**Background:** Glioblastomas are the most common and malignant central nervous system (CNS) tumors that occupied a highly heterogeneous tumor microenvironment (TIME). Long noncoding RNAs (lncRNAs), whose expression can be modified by DNA methylation, are emerging as critical regulators in the immune system. However, knowledge about the epigenetic changes in lncRNAs and their contribution to the immune heterogeneity of glioma is still lacking.

**Methods:** In this study, we integrated paired methylome and transcriptome datasets of glioblastomas and identified 2 robust immune subtypes based on lncRNA methylation features. The immune characteristics of glioma subtypes were compared. Furthermore, immune-related lncRNAs were identified and their relationships with immune evasion were evaluated.

**Results:** Glioma immunophenotypes exhibited distinct immune-related characteristics as well as clinical and epigenetic features. 149 epigenetically regulated (ER) lncRNAs were recognized that possessed inverse variation in epigenetic and transcriptional levels between glioma subtypes. Immune-related lncRNAs were further identified through the investigation of their correlation with immune cell infiltrations and immune-related pathways. In particular, the 'Hot' glioma subtype with higher immunoactivity while a worse survival outcome was found to character immune evasion features. We finally prioritized candidate ER lncRNAs associated with immune evasion markers and response to glioma immunotherapy. Among them, CD109-AS1 and LINC02447 were validated as novel immunoevasive biomarkers for glioma through *in vitro* experiments.

**Conclusion:** In summary, our study systematically reveals the crosstalk among DNA methylation, lncRNA, and immune regulation in glioblastomas, and will facilitate the development of epigenetic immunotherapy approaches.

## Introduction

Glioblastomas are the most common and lethal central nervous system (CNS) tumors around the world, which featured extremely aggressive and highly heterogeneous tumor immune microenvironment (TIME) 1. Increasing evidence suggests that immune therapy is a promising alternative approach for treating glioma tumors 2,3. However, the special TIME caused by the blood-brain barrier brings challenges to the application of immunotherapy. Recent studies have uncovered the complex of TIME in glioma tumors and identified valuable biomarkers related to immunotherapy response. For instance, the glioma subtype with the deficiency of NF1 drives macrophages/microglia infiltration, thus affecting the efficacy of therapeutic intervention 4. S100A4 was found to be sufficient to reprogram the immune landscape by regulating the immune suppressive T and myeloid cells in glioblastomas 5. However, the discovery of glioma immune subtypes has primarily focused on the transcriptional level of protein-coding genes (PCGs). Epigenetic features that contribute to glioma carcinogenic and immune heterogeneity are still completely unknown.

Aberration of DNA methylation, a fundamental feature of epigenetics, has a substantial impact on gene expression, further affecting the oncogenic progression of cancer 6. DNA methylation has been successfully utilized in the identification of CNS tumor biomarkers, subtype classification as well as immunotherapy efficacy prediction. For example, Capper et.al built a robust classifier based on DNA methylation to distinguish ~100 CNS tumors with discrete histo-molecular features 7. Combining DNA methylation will improve the prediction of glioblastoma with a poor prognosis than MRI classification 8. Genome-wide DNA methylation studies have also identified epigenetic markers that respond to immunotherapy in multiple cancer types such as melanoma and non-small cell lung carcinoma 9,10. These data highlight the pivot roles of DNA methylation in the classification of glioma and immune therapy response. However, crucial DNA methylation features to guide clinical treatment for glioma patients are still lacking.

Long noncoding RNA (lncRNA), a class of pervasively transcribed RNA molecules with lengths more than 200 nucleotides, is emerging as essential immune regulators that involved in carcinogenic processes 11. LncRNAs have been found to play important roles in the TIME of glioma tumors. HOXA-AS2 (HOXA Cluster Antisense RNA 2) was found to facilitate the expression of KDM2A/JAG1 to promote regulatory T cell proliferation and immune tolerance in glioma 12. HOTAIR (HOX Transcript Antisense RNA) could promote NF-kappaB phosphorylation and nuclear translocation by targeting NBXN1, thus induce immune escape for glioma patients 13. Meanwhile, the activities of lncRNAs were regulated by their promoter DNA methylation. Thousands of epigenetically regulated (ER) lncRNAs were found to be distributed in human tumors that associated with patient's survival 14. Therefore, further studies on the crosstalk among DNA methylation, lncRNAs, and immune regulation will be essential to identify novel immunotherapy targets in glioblastomas.

Here, we integrated methylome and transcriptome profiles of 551 glioma patients from the TCGA cohort to systematically identify the epigenetically regulated lncRNAs. We classified glioma patients into 2 optimal clusters based on lncRNA methylation, which were characterized by distinct immune and epigenetic phenotypes. Through assessing the association between immune cell infiltrations, immune pathways and lncRNAs, we identified a subset of immune-related lncRNAs with high confidence. Among them, CD109-AS1 (CD109 Antisense RNA 1) and LINC02447 (Long Intergenic Non-Protein Coding RNA 2447) are novel immune evasion markers in glioma, which were verified by *in vitro* experiments. These findings provide a theoretical basis for identifying epigenetic markers and developing strategies of multi-omics integration to guide clinical treatment for glioma patients.

## Methods

### Data collection and preprocessing

The Illumina HumanMethylation450 BeadChip (450K) and gene expression profiles for low-grade glioma (LGG) and glioblastoma multiforme (GBM) were downloaded from TCGA Pan-Cancer (PANCAN) cohort through UCSC Xena (https://xenabrowser.net/), which has performed batch effect correction. The patients with both methylome and transcriptome profiles (n = 551) were retained for further analysis. The clinical and molecular features include age, gender, grade, histology, isocitrate dehydrogenase (IDH) mutation, telomerase reverse transcriptase (TERT) mutation, alpha-thalassemia/mental retardation, X-linked (ATRX) mutation, 1p/19q codeletion, O^6^-methylguanine-DNA methyltransferase (MGMT) promoter methylation, tumor mutational burden (TMB), and chromosome aneuploidy of patients were collected from a previous study 15.

An external dataset of 59 glioma patients with paired 450K array and RNA-sequencing data was downloaded from the Gene Expression Omnibus (GEO) with accession number GSE121723. The expression levels of genes were measured as transcripts per million (TPM) and log2 transformed.

The segment of each brain sample for the 18-state Roadmap Epigenome ChromHMM model was downloaded from the Roadmap project 16. The CpG probes were mapped into different chromatin state regions through BEDTools 17 and averaged their beta values to quantify the methylation level.

### LncRNA annotations and methylation

The annotations of lncRNAs were downloaded from GENCODE (V34, GRCh38). The genomic locations of 450K probes were first transferred from GRCh37 to corresponding coordinates in GRCh38 through UCSC LiftOver (https://genome.ucsc.edu/cgi-bin/hgLiftOver). We then mapped the probes to the promoter region (4-kb regions centered at the TSS) for lncRNAs and protein-coding genes (PCGs) based on GENCODE V34 annotation. The methylation level was quantified by the averaged beta values of probes located in the promoter region.

### Identification of glioma subtypes based on lncRNAs methylation

We first selected the top 5% lncRNAs with high variation of methylation level (S.D. > 0.186). The ConsensusClusterPlus package was used to identify the glioma subtypes based on the DNA methylation profiles of lncRNAs 18. The procedure was run with 1,000 iterations and a sub-sampling ratio of 0.8. Only subtypes with more than 5 samples were retained.

### Likelihood Ratio Test

The Cox proportional hazards model was first constructed based on the gender and age information of each glioma patients. Then, clinical features including histology, 1p/19q codeletion, IDH mutation, MGMT methylation, TERT and ATRX mutation as well as our lncRNA methylation clusters were added to the models, respectively. We estimated the likelihood ratio (LR) statistic of these regression models and the changes in LR were assessed by Chi-square test.

### Identification of epigenetically regulated lncRNAs

The DNA methylation differences of lncRNAs between glioma subtypes were firstly compared based on Wilcoxon's rank sum tests. Differentially methylated lncRNAs were determined by the absolute value of methylation difference greater than 0.2 and FDR less than 0.05. The correlation between the methylation of lncRNA promoter and expression levels was further estimated by Pearson correlation analysis. Only differentially methylated lncRNAs with a negatively correlated with its expression were considered epigenetically regulated lncRNAs.

### Immune cell infiltration, immune signatures, and antitumor immunoactivity

Glioma tumor-infiltrating immune cell abundances were obtained from Tumor Immune Estimation Resource (TIMER) 19, which provided B cell, CD4+ T cell, CD8+ T cell, Neutrophil, Macrophage, and Dendritic cell.

The immune signatures gene sets, including immune checkpoints, human leukocyte antigen (HLA), tumor-infiltrating lymphocytes (TILs), and immune cell type-specific gene-CpG pairs for 21 immune cell populations were collected from one of our previous studies 20. In addition, gene sets for Macrophages/monocytes, Lymphocyte infiltration, TGF-β response, IFN-γ response, and Wound healing were collected from the previous study by Vesteinn et. al 21. The single-sample GSEA (ssGSEA) algorithm was applied to estimate the expression activity of immune signatures 22.

Antitumor immunoactivities were measured by three features as followed, (i) ESTIMATE score that reflects the tumor purity 23; (ii) MHC (Major histocompatibility class) score, which reflects the activity of antigen presentation, was calculated by the average gene expression of the MHC-I set 24; (iii) CYT (Cytolytic activity) score was computed as the geometric mean of the GZMA and PRF1, which denotes the cytolytic activity of immune cells against tumor cells 25. All gene sets related to immune signatures and immunoactivity are provided in Table S1.

### Immune evasion and immunotherapy response

The Tumor Immune Dysfunction and Exclusion (TIDE) algorithm was applied to estimate the immunotherapy response of glioma patients 26. Higher TIDE scores implicated a higher potential of tumor immune evasion, thus less likely to benefit from immunotherapy. KLRB1 (encoding CD161) mediated immunosuppression on T cells in glioma was enrolled to estimate the immune evasion character for glioma patients 27. The infiltration of regulatory T Cells (Tregs) which contribute to immune evasion of malignant gliomas was obtained from CIBERSORT-ABS 19. The anti-PD1 therapy of glioma patients (PRJNA482620) was also included for further analysis.

### Identification of immune-related lncRNAs

To identify immune regulatory lncRNAs involved in glioma subtypes, Pearson correlation analysis was firstly performed between expression/methylation levels of lncRNAs and immune cell infiltrating. In addition, 17 immune-related pathways were obtained from ImmPort project 28. Next, we applied ImmLnc algorithm to explore the relationship between lncRNA and immune-relate pathways 29. The method passed the ordered gene list which based on the partial correlation coefficients with a specific lncRNA to the GSEA algorithm with each immune-related pathway. LncRNAs with a FDR value less than 0.05 were considered as significant.

### Enrichment analysis

The functional analysis was performed based on the nearest protein-coding gene of lncRNA through Metascape 30. GSEA was also used to explore the dysregulation functions between glioma subtypes by R package “fgsea”. Additionally, eForge 2.0 was used to evaluate the enrichment of probes located in lncRNAs with DNA methylation alterations versus the presence of primed chromatin state histone modification marks 31.

### Cell culture and transfection

The human glioblastoma cell lines U251 and U87 used in this study were purchased from Procell Life Science&Technology Co.,Ltd. (Wuhan, China). All cell lines were grown in DMEM (Hyclone, USA) supplemented with 10% fetal bovine serum (FBS) (Gibco, USA) and incubated at 37 °C in a humidified atmosphere of 5% CO2.

Transfection of siRNA was performed using Hieff TransTM *in vitro* siRNA/miRNA Transfection Reagent (Yeasen, China) according to the manufacturer's instructions. U251 cells were plated in T-25 cells culture flasks and 96 wells plates, 24 wells plates, and transfected with siRNA. At 48-72 h after transfection, the cells were collected and used for experiments.

LncRNA CD109-AS1 cDNA was inserted into the pCDNA3.1 plasmid (CD109-AS1 -P; “P” representing plasmid). The plasmid vectors (CD109-AS1 -P and empty vectors as a negative control construct NC-P) were transfected into cells for CD109-AS1 overexpression at a final concentration of 2.5 mg/L.

### Cell scratch test

Cell scratch test was used to detect U251 cell migration. The digested cells of each group were seeded in 6-well plates (5×105/well) and cultured at 37 °C, 5% CO2 and 100% relative humidity until the cells reached about 90% confluence. Then cells were scratched from top to bottom. The scratched cells were washed and then were continued to culture for 24 h under the same conditions and observed under the microscope (Olympus). The moving distance of cell front and scratch width were measured to analyze the cell migration capability.

### Transwell assay

The transwell chamber (Corning) was placed in 24 well plates, then matrix glue was added to the transwell chamber, and the DMEM medium (10% FBS) was added to the bottom chamber. The digested and resuspended cells were seeded in the upper chamber with a density of 5×10^5^/chamber and then were cultured for 48 h. Subsequently, the medium was removed, cells were fixed with 4% polymethanol and stained with 0.1% crystal violet solution (Beyotime, China) for 10 min. The number of cells invading the basal chamber in each field was calculated under the microscope (Olympus).

### CCK-8 assay

U251 cells were seeded at 5×103 cells per well in 100 μL DMEM medium in 96-well plates and incubated at 37 °C (5% CO2) for 24 hours to the logarithmic growth period, 24, 48, and 72 h after culture, and then 10 μL CCK-8 reagent was added (Beyotime, Beijing, China), and then cells were cultured in the original medium for 2 h. After that, the absorbance at 450 nm was calculated for cell viability.

### Western blotting

Total protein was extracted using RIPA lysis buffer with a protease inhibitor cocktail. The protein concentrations were normalized with a BCA assay kit (Beyotime, China). Proteins from each group were loaded onto SDS-PAGE gels and separated before they were transferred to PVDF membranes (Millipore, USA). The membrane was incubated with primary antibodies against CTLA4 (1:1000, Ag24178; Proteintech, China), FOXP3 (1:1000, 22228-1-AP; Proteintech, China), PD-L1 (1:1000, 28076-1-AP; Proteintech, China), MHC-I (1:1000, PTM-6400; Biolab, China), and GAPDH (1:1000, 14C10; Cell Signaling Technology) at 4 °C overnight. Afterward, the membrane was incubated with a secondary antibody at room temperature for 2 h, the blotting was developed by using the ECL (Beyotime, China) plus immunodetection system.

### Quantitative real-time PCR (qRT-PCR)

The transfected treated U251 were cultured for 48 h and the cells were collected, the expression levels of IncRNA AC131097.3, CD109-AS1, LINC02447 and LINC01765 were determined by qRT-PCR, and GAPDH was used as the internal control. In brief, total RNA was extracted using TRIzol reagent (50 mg/mL) according to the manufacturer's protocol (Transgen, China). Reverse Transcription and quantitative RT-PCR was performed with a cDNA synthesis kit (Yeasen, China) according to the manufacturer's instructions. Amplification in a bio-rad CFX96 real-time PCR system was conducted as follows: 5 min at 95 °C, followed by 40 cycles of 10 s at 95 °C, 55-60 s at 20 °C and 20 s at 72 °C. The nucleotide sequences of the primers used for qRT-PCR are shown in Table S2. Gene expression levels were recorded as threshold cycle (Ct) values. Each sample was amplified twice. A melting curve analysis was performed on each sample to ensure single amplification.

### Statistical analysis

R software 4.1.0 was conducted in this study for statistical analyses. Correlation analysis was performed using the Pearson correlation test. Wilcoxon rank-sum test was used to estimate the differences between glioma subtypes. R package “edgeR” was used to perform differently expressed genes (DEG) analysis. The p-value was adjusted by the FDR method and all tests with FDR < 0.05 were considered to be statistically significant. Survival analysis was visualized using the Kaplan-Meier curves by R package “Survminer”. Stepwise multivariate Cox hazard regression was performed by R package “MASS”. Experimental data are expressed as mean ± SEM and they were analyzed with SPSS 24.0 software. Statistical comparisons among multiple groups were performed using analysis of variance (ANOVA) followed by Dunnett's test. Student t-test was carried out for comparisons between the two groups. Chi-Square test was used for nonparametric data set comparisons.

## Results

### LncRNA methylation heterogeneity reveals glioma immunophenotypes

**Figure 1:**
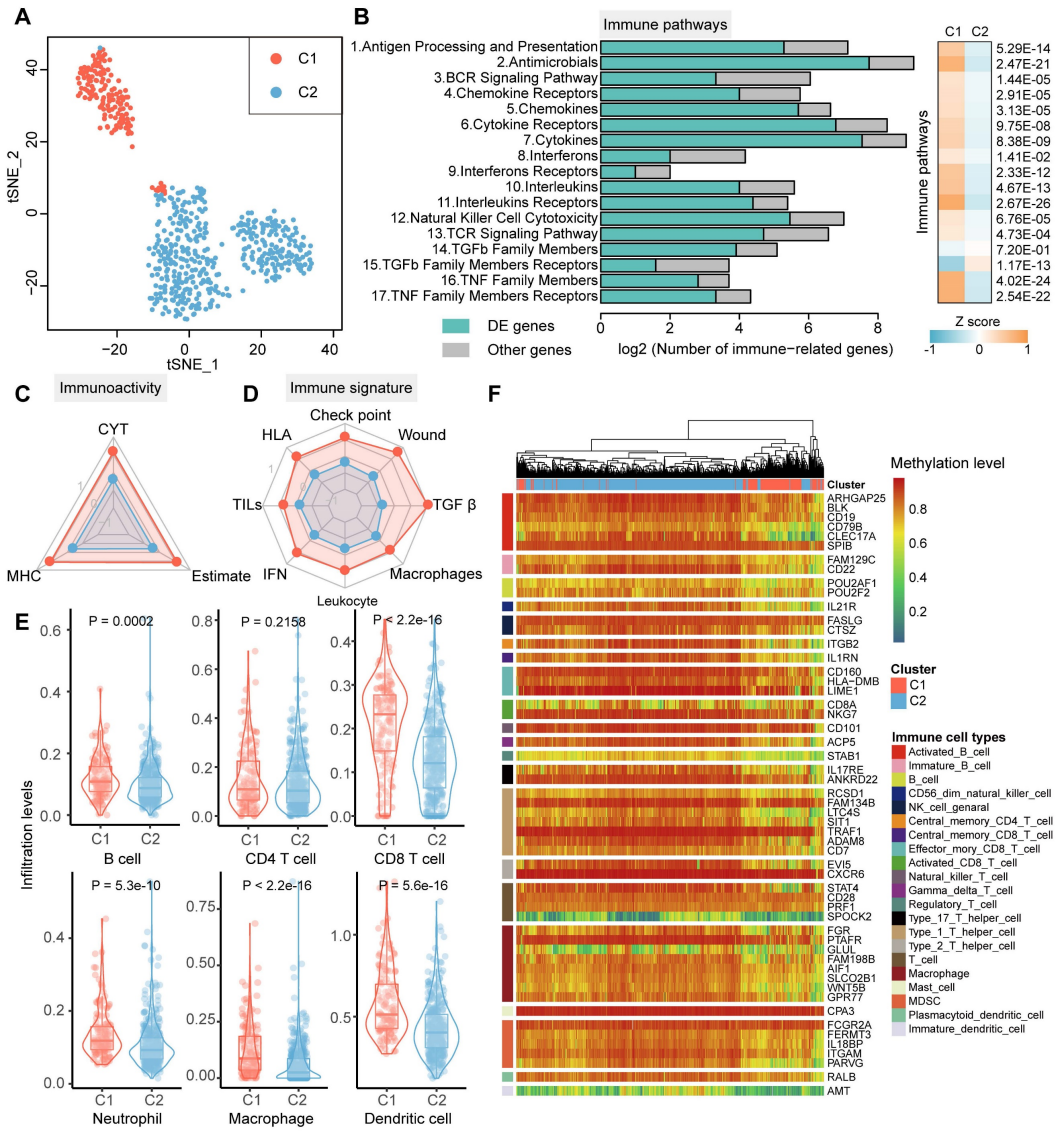
** Characteristics of tumor immunophenotypes in glioma via lncRNA methylation.** (A) tSNE dimensionality reduction plot of glioma samples using lncRNA methylation as features. Individual samples are colored by the subtypes. (B) Bar plots showing the number of DE genes between glioma subtypes in each immune-related pathway. The green bars are for DE genes and gray bars are for other genes. Right-side heat map showing the difference of immune pathway activities estimated by ssGSEA between glioma subtypes. (C) and (D) The mean value of scaled antitumor immunoactivity and immune signature scores between glioma subtypes. Points colored by red and blue represents C1 and C2 cluster, respectively. (E) Violin plots showing the levels of immune cell infiltrations obtained from TIMER for glioma subtypes. (F) Heat map showing the relative methylation of immune cell type marker genes in different glioma subtypes.

DNA methylation facilitates the depiction of histopathological diagnosis and immune heterogeneity for many CNS tumors 7,32. Hence, 551 glioma patients with both DNA methylome and transcriptome profiles from TCGA cohort were selected to investigate the extent of the lncRNA methylation heterogeneity. Tumors were subtyped into 2 optimal clusters (C1 and C2) through consensus clustering of lncRNAs with variable DNA methylation (Figure S1). We constructed the tSNE embedding clustering to visualize the clusters with lncRNA methylation features, where C1 and C2 were separated in two dimensions (Figure 1A). Additionally, the patients in the C1 cluster were from both GBM and LGG, while C2 was mainly formed by LGG patients (Figure S1).

To investigate whether the difference in lncRNA methylation pattern implied intertumoral immune heterogeneity and microenvironment, we next focused on the 17 immunologically relevant pathways derived from ImmPort 28. DEG analysis results showed that large proportion of genes in immune pathways exhibited expression dysregulation between glioma clusters (|logFC| > 0.75, FDR < 0.05, Figure 1B). Apart from TFGβ family members and receptors, the activity of the rest immune pathways were significantly higher in C1 cluster based on ssGSEA of the genes expression (Wilcox.test P < 0.05, Figure 1B). Moreover, we estimated the antitumor immunoactivity scores for glioma patients, including CYT, MHC and ESTIMATE score. All the three immunoactivity scores were significantly increased in C1 cluster, indicating patients in C1 cluster displayed greater tumor-killing activity, stronger antigen presentation capacity and lower tumor purity (Figure 1C). In addition, C1 cluster exhibit higher activities of immune signatures (Figure 1D). For instance, higher IFN score were observed in C1 cluster, which implies that C1 were more able to suppress tumor growth 33. We next assessed the relative proportion of immune cells of glioma patients using TIMER. Similarly, the infiltrate levels for B, CD8 T, neutrophil, macrophage and dendritic cells were significantly elevated in C1 cluster (Figure 1E). Therefore, we summarized the C1 cluster was attributed to immune 'Hot' phenotype, while C2 was characterized as immune 'Cold' phenotype according to the above evidence. We also found the marker genes of multiple immune cells exhibited diverse DNA methylation patterns between clusters, where patients in C1 showed hypo-methylated and C2 displayed hyper-methylated (Figure 1F). Collectedly, these results suggested the heterogeneity of DNA methylation (especially lncRNA) could reveal glioma immunophenotypes.

### Glioma immunophenotypes carried distinct clinical and epigenetic features

**Figure 2:**
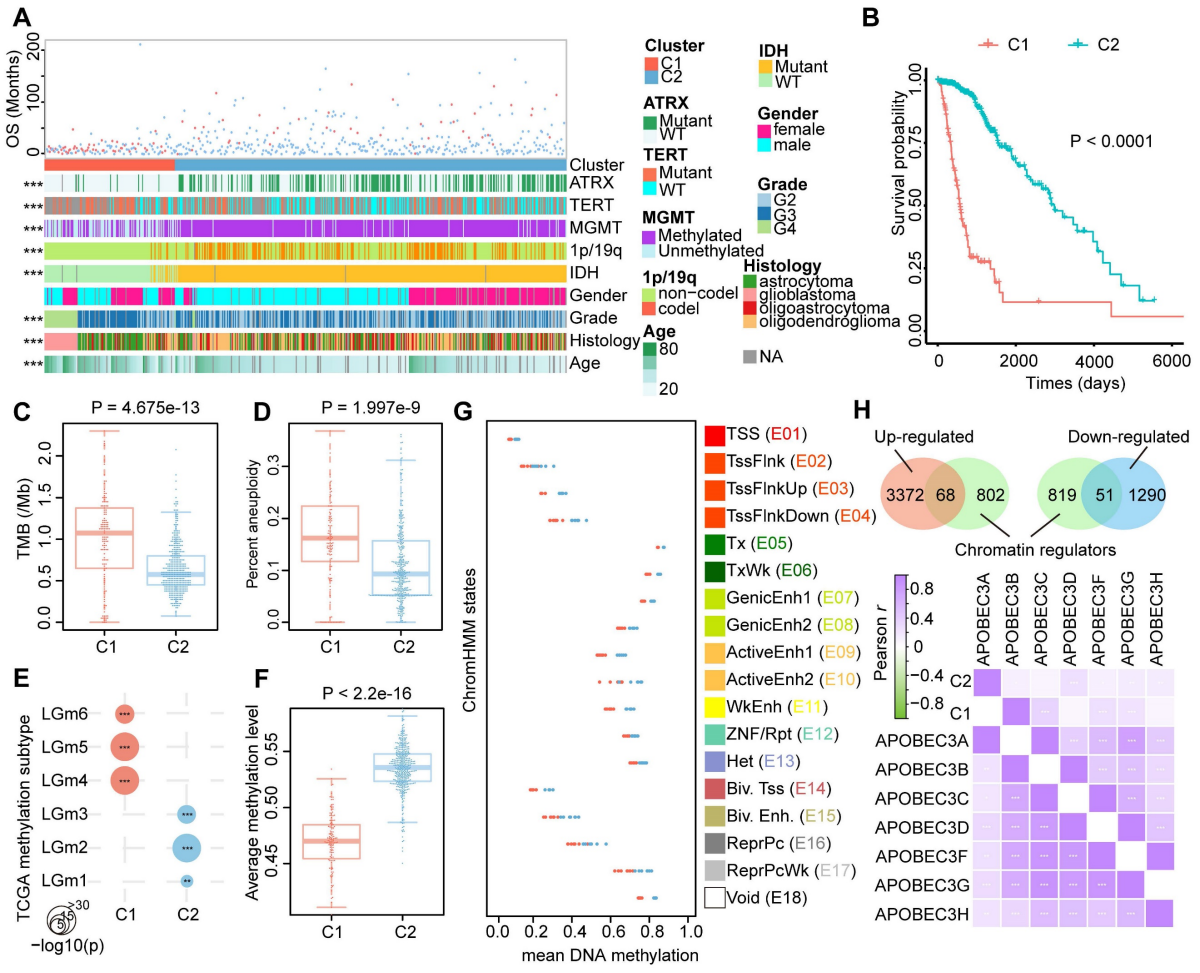
** Clinicopathological and epigenetic characteristics of glioma subtypes.** (A) The landscape of clinicopathological features of glioma samples in TCGA cohort. The significance of the difference was tested by the chi-square test. (B) The Kaplan-Meier plot of glioma patients based on lncRNA methylation classifications. (C) and (D) The differences of molecular features (TMB and aneuploidy score) for glioma subtypes. (E) Bubble plot showing the enrichment between glioma immunophenotypes and TCGA well-defined subtypes based on DNA methylation. (F) The difference of average methylation levels for lncRNAs between glioma subtypes. (G) Mean subtype DNA methylation level for each state of the 18-state ChromHMM model. (H) Venn plot showing the overlap between DE genes and chromatin regulators. Bottom-side heat map showing the co-expression of APOBEC3 family in glioma subtypes.

Cancer subtype classification with distinct clinical and molecular features has shed light on the understanding of the mechanisms driving gliomagenesis 15. To investigate whether the glioma immunophenotypes carried unique clinical pattern, we next conducted comparative analysis based on several essential molecular and clinical features. We found that ATRX as well as TERT mutation status, MGMT promoter methylation status, 1p/19q codeletion status, IDH mutation status, WHO grade, histology diagnosis, and the age of patients exhibited asymmetric distributions (Figure 2A, Chi-squared test P < 0.05). Upon comparison of the survival rates, we found C1 patients had worse prognosis than C2 patients (Figure 2B, log-rank test P < 0.05).

The ratio of alive patients was increased in the C2 cluster, whereas a large proportion of dead patients was assigned to C1 cluster (Figure S2A). Multivariate Cox proportional hazard analysis indicated the prognostic independence of the glioma classifications based on lncRNA methylation was behind the grade and age when considering the clinical features mentioned-above (Figure S2B). Stepwise multivariate Cox hazard regression showed that the grade, age, TERT mutation, and our subtypes were tended to be the independent risk factors for glioma survival, with the cluster based on lncRNA methylation being the most significant factor (Figure S2C). Moreover, we observed a large increase in the predictive fit by considering the lncRNA methylation than other clinical features (only worse than IDH mutation), implying the clinical value of lncRNA methylation in glioma (Figure S3). By calculating TMB in the coding region (total number of mutations per sample / 40Mb) and chromosome aneuploidy, we observed an overall high level of these two features in C1 cluster (Figure 2C and 2D). Except for the genetic variations, the epigenetic alterations were also explored. Diffuse gliomas have been subtyped into six discrepant clusters (LGm1-LGm6) based on DNA methylation in TCGA cohorts 15. We found C1 cluster was enriched for LGm4-6 with genome-wide hypo-methylation, while LGm1-3 were enriched for our C2 cluster (Figure 2E, hypergeometric test P < 0.05). Notably, there was a significant DNA hypomethylation level of lncRNAs in C1 than that in C2 cluster (Figure 2F).

Next, the DNA methylation levels between glioma clusters were compared using the 18-state Roadmap Epigenome ChromHMM model. The 450K probes were firstly annotated to the 18 chromatin states through BEDTools and calculated their mean methylation level for each brain samples in Roadmap. Similar with previous study, active TSS states (E01-04), the bivalent TSS as well as enhancer states (E14-15) exhibited relatively hypomethylation, while transcription (E05-06), repressive (E12-13, E16-17) and non-functional (E18) states have higher methylation (Figure 2G) 34. Generally, the patients of C1 cluster have lower methylation levels than C2 cluster in all 18 chromatin states. Since chromatin states of human genome were regulated by chromatin regulators (CR), we next estimated the expression patterns of these molecules derived from a recent study 35. In total, 119 CRs (including 69 up-regulated and 51 down-regulated) were identified as differently expressed (Figure 2H). Particularly, most of the APOBEC family, a class of 5mC or 5hmC deamination mediators, were found to be up-regulated and co-expressed in C1 cluster (Figure 2I and Figure S4) 36. These results indicated that the global hypo-methylation of glioma C1 patients was regulated by APOBEC family.

### Epigenetically regulated lncRNAs are related to cancer processes

**Figure 3:**
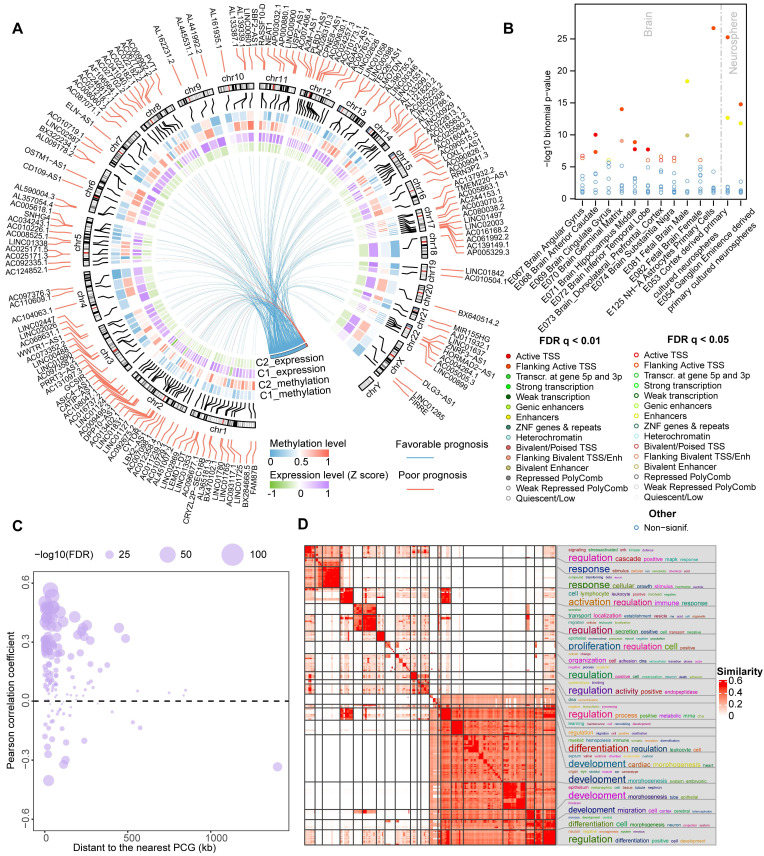
** Epigenetically regulated lncRNAs were correlated with glioma development.** (A) Circos plot showing the 149 ER lncRNAs in glioma subtypes. The inner heat map shows the mean methylation and expression levels of lncRNAs in glioma immunophenotypes. Red and blue lines represent the methylation of lncRNA associated with a poor and better prognosis for glioma patients. (B) The enrichment between CpGs probes belonging to the promoter of ER lncRNAs and 15 chromatin states regions for brain samples via the eForge tool. (C) Relationships between ER lncRNAs expression and nearest PCG expression. (D) Functional enrichment analysis of nearest PCG of ER lncRNAs.

DNA methylation, a fundamental feature of epigenetics, plays a pivotal role in regulating the activities of lncRNAs 14. We next combined expression and methylation data to identify epigenetically regulated lncRNAs in glioma patients. In total, we found that 149 ER lncRNAs exhibited a negative correlation between their expression and promoter DNA methylation levels (Figure 3A, Table S3). These ER lncRNAs also possessed varied DNA methylation level between C1 and C2 clusters (|delta beta| > 0.2, Wilcox.test FDR < 0.05). Most ER lncRNAs (144/149) showed hypo-methylated in the promoter region with their expression increased in C1 patients compared to the C2 cluster. Notably, all the methylation of ER lncRNAs could predict the prognosis of glioma patients based on their DNA methylation level (Figure 3A, Table S3). We next used the most 1000 hypo-methylated probes located in the promoter region of ER lncRNAs to perform eForge analysis 31. The CpG sites were related to active TSS chromatin states (i.e. E1 Active TSS) and histone modifications associated with transcriptional activation (i.e. H3K4me1) of brain samples from Roadmap (Figure 3B and Figure S5), which further proved the activated expression of lncRNAs by the DNA methylation. To explore the potential biological functions of ER lncRNAs, we assigned the lncRNAs to their nearest protein-coding genes (PCGs) and performed a co-expression analysis. Approximately 70% lncRNA-PCG pairs were significantly correlated between their expression level (Figure 3C and Figure S6). Functional enrichment analysis revealed that PCGs regulated by ER lncRNAs were significantly enriched in immune regulation and nervous system developmental processes (Figure 3D). Moreover, there were 83.22% ER lncRNAs exhibited expression deviations between different glioma grades and histology (119 lncRNAs for grades and 118 lncRNAs for histology, Table S3, ANOVA tests P < 0.05). Together, these observations suggested that the activities of lncRNA were regulated by DNA methylation and strongly related to glioma processes.

### Epigenetically regulated lncRNAs associated with immune regulation

**Figure 4:**
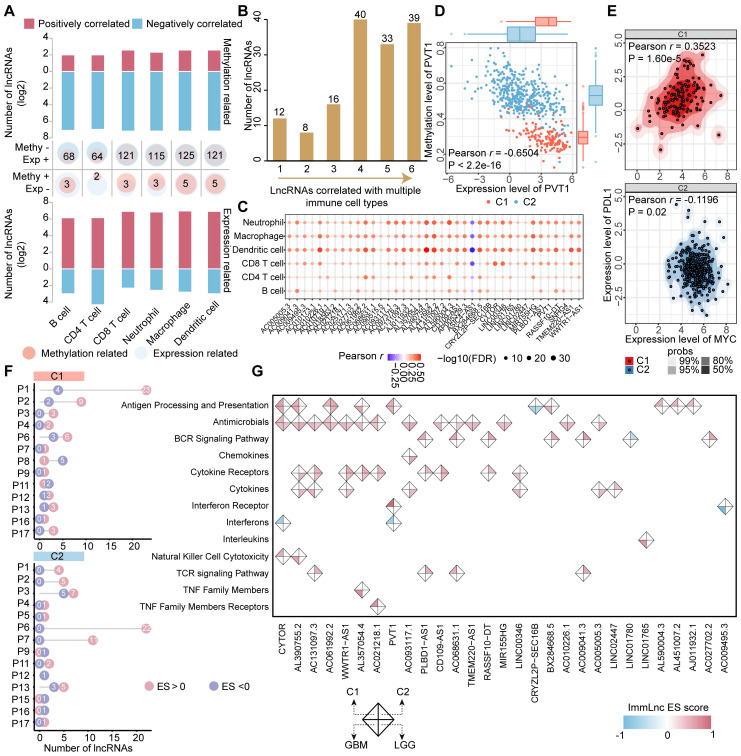
** Epigenetically regulated lncRNAs involved in immune regulation.** (A) Bar plots showing the number of lncRNAs in which methylation and expression correlated with immune cell infiltrations. Venn plots display the number of lncRNAs that correlated with specific immune cell infiltration with different directions in expression and methylation levels. (B) The number of lncRNAs that correlated with different numbers of immune cell types. (C) Balloon plots of correlation between lncRNA expression and immune cell infiltration. (D) Correlation between the methylation and expression level for PVT1. (E) Correlation between the expression of MYC and PDL1 in different glioma subtypes. (F) The distribution of identified immune-related lncRNAs across immune pathways in different glioma subtypes. (G) The landscape of essential ER lncRNAs involved in the regulation of immune pathways.

LncRNAs are emerging as critical immune regulators that involve in the stabilization of TIME 37. We next evaluated the correlation between the methylation/expression level of ER lncRNAs and immune cell infiltrations in each cluster of glioma patients. Only pairs that satisfy the relationship that expression and methylation have opposite directions are retained. In total, we identified 66 to 130 lncRNAs significantly associated with infiltrating immunocytes in both methylome and transcriptome levels (Figure 4A). The number of positively correlated lncRNAs was higher than that of negatively correlated ones in expression level. In particular, there were 39 lncRNAs correlated with all 6 immune cell infiltrations (Figure 4B). Interestingly, we found that the immune cell infiltrations were positively regulated by almost ER lncRNAs, only ASIC4-AS1 exhibited negative correlations (Figure 4C). To further investigate the immune regulation of ER lncRNAs, we took a hot immune-related lncRNA PVT1 as an example. We found that PVT1 was hypo-methylated, thus elevating its expression in glioma C1 patients (Figure 4D). PVT1 has been reported to interact with the nearest protein-coding gene MYC and activate its downstream molecules to synergistically promote tumorigenesis 38. As the downstream target, PD-L1 was positively correlated with MYC in the cluster with high PVT1 expression and immune cell infiltration (C1, Figure 4E). These results were consistent with observations that the PD-L1 was involved in the recruitment of T cells and macrophages and modified the TIME 39. Together, the hypo-methylated lncRNA might activate the expression of lncRNA first, further influence the target and downstream genes, and finally regulate the immune cell levels.

We next estimated the associations of ER lncRNAs and 17 categories of immune pathways through a previous computational framework 29. The distribution of ER lncRNAs related to immune pathways was diverse between glioma clusters. For instance, 23 ER lncRNAs were positively associated with antigen processing and presentation pathways, while 22 ER lncRNAs were enriched in the cytokine receptors (Figure 4F). We further focused on the 39 ER lncRNAs that correlated with all infiltrating immunocytes in glioma. There were 29 lncRNAs involved in the regulation of immune pathways. Consistent with the immune cell infiltration, most lncRNAs were positively associated with immune pathways (Figure 4G). Among them, the positively correlated PCGs of PVT1 were enriched in antigen processing and presentation as well as interferon receptor pathways, while negatively correlated PCGs enriched in interferons in the C1 cluster (Figure S7). Taken together, these results suggest that ER lncRNAs were associated with immune cell infiltration and immune regulation in glioma.

### ER lncRNAs associated with glioma immune evasion

**Figure 5:**
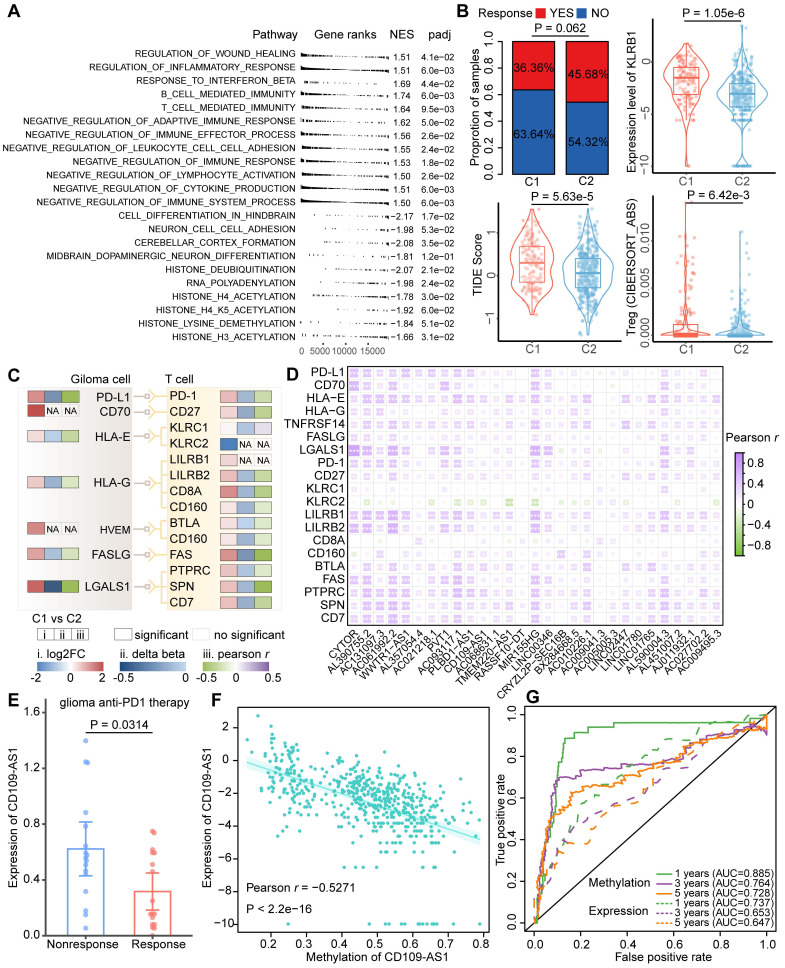
** Identification of ER lncRNAs serves as novel immunoevasive markers.** (A) Gene Ontology (GO) analysis of DE genes between glioma immunophenotypes. (B) Immune evasion scored estimated by the TIDE tool. Bar plot showing the proportions of patients who responded to immunotherapy in glioma subtypes. Box plot showing the differences of TIDE score, KLRB1 expression, and Tregs infiltrations between glioma subtypes. (C) The expression, methylation patterns as well as correlations between expression and methylation of the well-known immune evasion markers for glioma. (D) Correlation between glioma immune evasion markers and ER lncRNAs. (E) The expression level of lncRNA CD109-AS1 in response and nonresponse glioma patients received anti-PD1 immunotherapy. (F) Correlation between the expression and methylation level of CD109-AS1 in TCGA glioma cohort. (G) Comparison of prognosis efficacy between methylation and expression of CD109-AS1.

The C1 cluster was characterized as an immune 'Hot' phenotype, yet patients in this cluster had a worse prognosis than those in the C2 cluster. To explore the probable cause of this contradictory phenomenon, PCGs were ordered based on the fold change of their expression between glioma clusters and performed GSEA functional analysis. Apart from immune regulation processes, multiple immunosuppressive gene sets were enriched in the C1 cluster, while brain developmental processes and histone modifications were enriched in the C2 cluster (Figure 5A). Through TIDE analysis, we found patients in the C1 cluster had lower efficacy of immunotherapy treatment compared to those in the C2 cluster (Figure 5B). Moreover, the TIDE score, KLRB1 expression, and Tregs infiltrations in the C1 cluster were significantly increased, indicating the patients in the C1 cluster may occurred immune evasion (Figure 5B). We thus corrected cell surface markers expressed by GBM cells and corresponding receptors related to T cell immunosuppress from a previous study, and further estimated their expression and methylation level between glioma clusters 40. Most of the immune escape markers were hypo-methylated, hence up-regulated in the C1 cluster (Figure 5C), which is consistent with a recent study that the widespread hypomethylation elicits an immune evasion program in GBM cells 41.

To investigate the associations between ER lncRNA and glioma immune evasion, we further performed a Pearson correlation analysis between the expression of 29 immune-related lncRNAs (related to both immune cell infiltration and immune pathways) and immunosuppressive markers. A global positive correlation was observed in glioma patients (Figure 5D). By analyzing a dataset of anti-PD1 therapy for glioma, we found the expression of AC131097.3, AL590428.1 (also known as CD109-AS1 in GENCODE V41), LINC02447, and LINC01765 were significantly decreased in the patients with immune therapy response (Figure 5E and Figure S8). The expression levels of these ER lncRNAs were negatively correlated with their promoter methylation (Figure 5F and Figure S9). In addition, low methylation and high expression of the four lncRNAs were associated with worse survival of glioma patients (Figure S10). Similar to the result of the previous study 42, the ER lncRNA methylation predicts the prognosis better than expression data (Figure 5G and Figure S10). Consistent with these findings, a negative association between DNA methylation and three ER lncRNA (AC131097.3, CD109-AS1 and LINC02447) expression as well as their prognosis capacities were observed in other glioma datasets with paired methylome and transcriptome (Figure S11). Taken together, these results suggested that lncRNA hypo-methylation might activate their expression, increased the risk of prognosis, and resisted immunotherapy.

### Validation of the association between ER lncRNAs and immune evasion *in vitro* experiments

**Figure 6:**
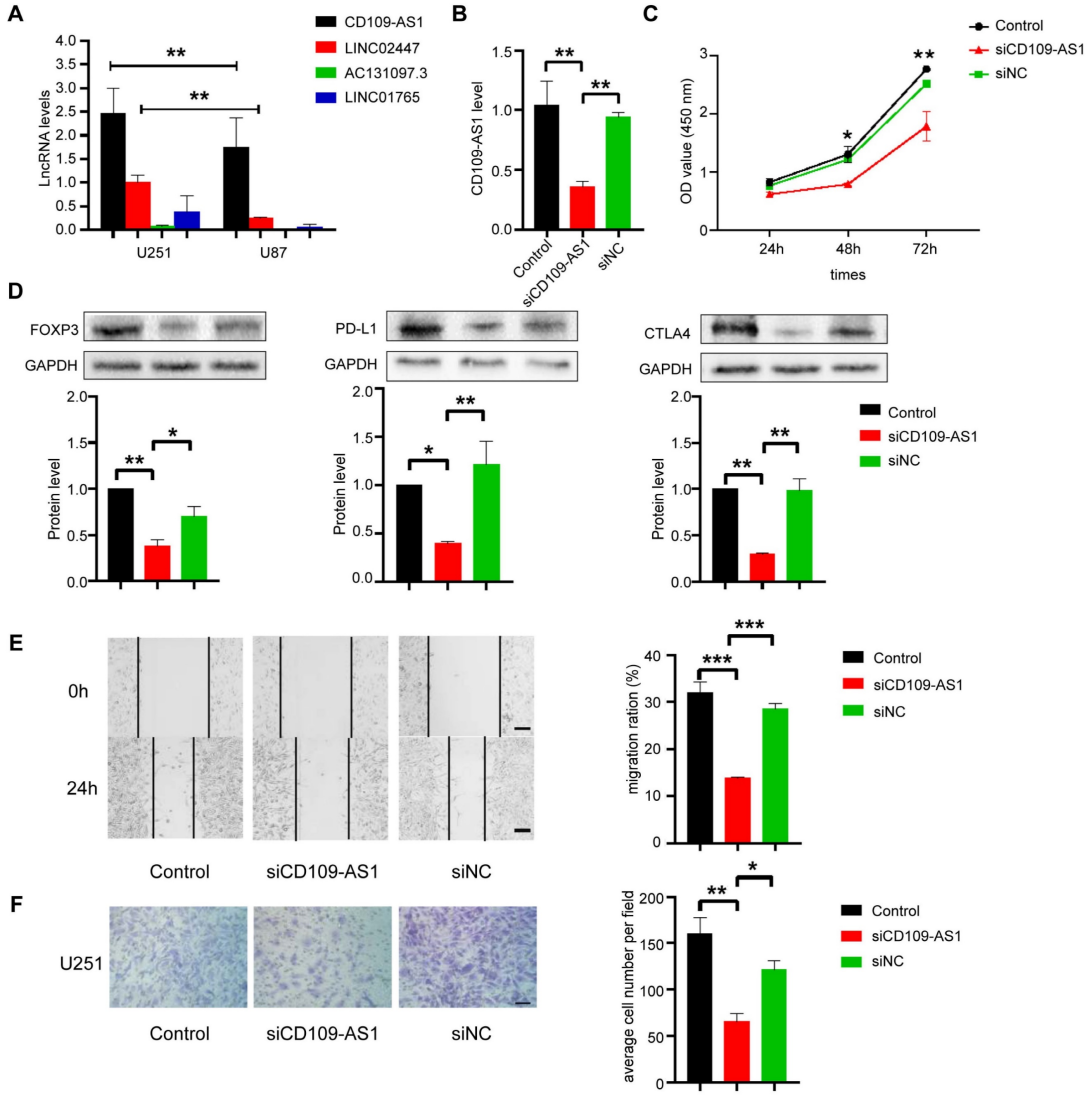
** Role of CD109-AS1 in the proliferation, migration, invasion and immune evasion of GBM cell lines.** (A) The expression of ER lncRNAs (AC131097.3, CD109-AS1, LINC02447 and LINC01765) in U87 and U251 cells was determined by qRT-PCR. (B) The expression of CD109-AS1 in U251 cells transduced with siRNA was examined by qRT-PCR (n = 3-5, **p < 0.01). (C) CCK-8 assays show that the inhibition of CD109-AS1 decreased cell proliferation in U251 cell lines cells. (n = 6, *p < 0.05, **p < 0.01). (D) The expression of FOXP3, CTLA-4 and PD-L1 in U251 cells transduced with CD109-AS1 siRNA was examined by Western blot; GAPDH was an internal control (n = 3, *p < 0.05, **p < 0.01). (E) Wound healing assays show that CD109-AS1 knockdown significantly reduced the cell migration ability of U251 cells with the representative images on the left and the quantitative analysis on the right (n = 3, ***p < 0.001). (F) Transwell invasion of CD109-AS1 siRNA GBM cells is significantly reduced compared with control cells (n = 3, **p < 0.01).

The capacities of ER lncRNAs as novel immune evasion markers for glioma were next validated *in vitro* experiments. We first detected the expression level of the four ER lncRNAs (AC131097.3, CD109-AS1, LINC02447, and LINC01765) in two GBM cell lines (U251 and U87) by qRT-PCR assays, and found that CD109-AS1 and LINC02447 were highly expressed in both cell lines while the AC131097.3 and LINC01765 were shown to undergo deletion (Figure 6A). Thus, siRNA technology was used to knock down the CD109-AS1 and LINC02447 in the U251 cell line and measure the expression of lncRNAs. We found that siRNA significantly reduced the expression of lncRNAs compared with un-transfected parental cells, indicating an effective lentivirus-delivered siRNA sequence (Figure 6B and Figure S12A). We further performed CCK8 to investigate the effect of CD109-AS1 and LINC02447 on the viability of GBM cells. Silencing of ER lncRNAs delayed the proliferation of U251 cell lines (Figure 6C and Figure S12B). Moreover, the knockdown of ER lncRNAs (CD109-AS1 and LINC02447) significantly inhibited the well-known immune evasion markers which included PD-L1, CTLA4, and FOXP3 in the protein level (Figure 6D and Figure S12C). Wound healing and transwell assays were employed to explore whether cell migration and invasion were influenced after silencing of ER lncRNAs. We found that the knockdown of ER lncRNAs significantly decreased cell migration and invasion compared with the control. (Figure 6E, 6F and Figure S12D, S12E). In contrast, the overexpression of CD109-AS1 significantly increased the expression of PD-L1, FOXP3, CTLA4 as well as cell migration and invasion (Figure S13). It has been reported that the loss of MHC-I will facilitate tumor immune evasion by affecting the antigen presentation process 43. The knockdown of CD109-AS1 and LINC02447 also rescued the protein levels of MHC-I (Figure S14). Taken together, these results suggested that CD109-AS1 and LINC02447 significantly promote in vitro cell migration, invasion, proliferation, and are associated with immune evasion in glioma.

## Discussion

In this study, using paired lncRNAs methylome and transcriptome analyses, we identified glioblastoma subtypes with distinct immune landscape which was significantly reflected in immune pathways, immunoactivity, signatures as well as TIME. However, the performance of mRNA methylation is not superior in classifying glioma patients (Supplementary Figure S15). The glioma subtypes based on lncRNA methylation also exhibited disparate clinical features and methylation levels in 18 chromatin states. Particularly, we found the expressions of APOBECA~H, a subset of DNA methylation eraser, were all increased in the immune-hot subtype accompanied with the genome-wide demethylation of this cluster. Moreover, the fold changes of APOBEC famaliy were ranked top compared to other DNA methylators, implying the essential roles of these gene family in glioma subtypes (Figure S16A). When considered all the differentially expressed CRs, two co-expression modules were identified in glioma patients, and the co-regulated relationships among APOBEC famaliy were also observed (Figure S16B). Our results are in accordance with the facts that APOBEC family could mediate deamination and initiate an active process of demethylation in human cells 36.

An increasing number of studies reveal that the variations of DNA methylation in the promoter region of lncRNAs could affect their activities and control the extent of interaction with target genes 14,44. Therefore, we simultaneously considered the change of expression and methylation level of lncRNAs between glioma subtypes and identified 149 ER lncRNAs. Similar to the global demethylation in the C1 cluster, 96.64% of ER lncRNAs (144) exhibited hypo-methylated thus their expression upregulated than the C2 cluster. Notably, ER lncRNAs were found to be associated with the survival of glioma patients, and the CpG probes of ER lncRNAs were enriched in the active signal of chromatin states and histone modifications. Thus, we indeed found serious lncRNAs that are regulated by DNA methylation between glioblastoma immune-related subtypes. Moreover, the genes regulated by ER are significantly enriched in brain development and immune systems.

LncRNAs are emerging as essential regulators involved in the immune systems and play critical roles in the treatment of cancer immunotherapy 45,46. Therefore, it is necessary to determine the relationship between our ER lncRNAs and immune regulation, so we identified immune-related lncRNAs by estimating their correlations with infiltrating immunocytes and immune pathway activities. Using this strategy, 29 essential ER lncRNAs were prioritized, of which 7 were reported to be associated with immune regulation and glioblastoma therapy from previous studies (i.e. PVT1, MIR155HG, and LINC00346). For instance, PVT1 has been found to regulate the immunosuppression activity of myeloid-derived suppressor cells (MDSCs), which inhibit the cytotoxic responses mediated by natural killer cells and blocked T cell-induced antitumor responses in glioma 47,48. Another example is MIR155HG, which has been reported to be highly expressed in GBM patients compared with LGG and normal brain tissues and involved in the extracellular matrix and response to wounding 49. These results suggest that the integration of DNA methylation and transcriptome is an effective method to study the immune regulation of lncRNAs.

Glioblastoma has been reported to feature intrinsic properties of immune evasion with the specialized immune context of the brain 50. Since C1 clusters displayed a high level of immune cells Infiltration and poor survival rates, we determined the C1 cluster as an immunosuppressive subtype and verified through the combination of functional enrichment analysis, TIDE algorithm, KLRB1 expression, Treg infiltration, and several glioma immune evasion markers. KLRB1 (encoding CD161) has been reported as an inhibitory receptor for glioma immunotherapy, whose blockade will enhance T cell-mediated killing of glioma cells both *in vitro* and *in vivo* experiments 27. Apart from the typical PD-1/PD-L1 pair involved in glioma immune evasion, glioma cells were also found to express non-classical MHC class I molecules on their surface to evade immune killing. For instance, the binding between HLA-G and CD8A on T cells will induce a Fas-FasL mediated apoptosis of CD8+ T cells 51. Moreover, the interactions of CD70, HLA-E, HVEM, FALSG, and LGALS1 expressed on the glioma cell surface and their corresponding receptors have been reported to cause T cell dysfunction, thereby enabling glioma cells to evade immune-mediated killing. Besides TIGIT and LAG3, the expressions of the rest of the T cell exhaustion markers including HAVCR2, CTLA4, PDCD1, and LAYN were significantly increased in the C1 cluster (Wilcox.test P < 0.05, Figure S17). The globally upregulated of these essential genes and ligand-receptor pairs in the C1 cluster further proves the immune evasion features of these patients. We found almost 29 essential ER lncRNAs were correlated with the expression of immune evasion markers. Among them, the potential roles of CD109-AS1 and LINC02447 in immunosuppression were both verified in anti-PD1 therapy and *in vitro* experiments, which provided novel targets for glioma immunotherapy.

In summary, our study systematically characterized the immune subtypes for glioma patients based on lncRNAs methylome and depicted the crosstalk among DNA methylation, lncRNA, and immune regulation. Through bioinformatics analysis and experiment verifications, we were the first to find and validated two lncRNA biomarkers, CD109-AS1 and LINC02447 involved in immune evasion, which will facilitate the development of immunotherapeutic targets for glioblastoma.

## Supplementary Material

Supplementary figures.

Supplementary tables.

## Abbreviations

CNS: central nervous system
TIME: tumor microenvironment
lncRNAs: long noncoding RNAs
ER: epigenetically regulated
PCG: protein-coding gene
CD109-AS1: CD109 Antisense RNA 1
LINC02447: Long Intergenic Non-Protein Coding RNA 2447
LGG: low-grade glioma
GBM: glioblastoma multiforme
IDH: isocitrate dehydrogenase
TERT: telomerase reverse transcriptase
MGMT: O6-methylguanine-DNA methyltransferase
TMB: tumor mutational burden
TPM: transcripts per million
TIMER: Tumor Immune Estimation Resource
HLA: human leukocyte antigen
TILs: tumor-infiltrating lymphocytes
ssGSEA: single-sample GSEA
MHC: Major histocompatibility class
CYT: Cytolytic activity
TIDE: Tumor Immune Dysfunction and Exclusion
Tregs: regulatory T Cells
qRT-PCR: Quantitative real-time PCR
DEG: differently expressed genes
CR: chromatin regulators

## References

[1] Nicholson JG, Fine HA (2021). Diffuse Glioma Heterogeneity and Its Therapeutic Implications. Cancer Discov [Internet].

[2] Yang K, Wu Z, Zhang H (2022). Glioma targeted therapy: insight into future of molecular approaches. Mol Cancer [Internet].

[3] Lim M, Xia Y, Bettegowda C, Weller M (2018). Current state of immunotherapy for glioblastoma. Nat Rev Clin Oncol [Internet].

[4] Wang Q, Hu B, Hu X (2017). Tumor Evolution of Glioma-Intrinsic Gene Expression Subtypes Associates with Immunological Changes in the Microenvironment. Cancer Cell [Internet].

[5] Abdelfattah N, Kumar P, Wang C (2022). Single-cell analysis of human glioma and immune cells identifies S100A4 as an immunotherapy target. Nat Commun [Internet].

[6] Nishiyama A, Nakanishi M (2021). Navigating the DNA methylation landscape of cancer. Trends Genet [Internet].

[7] Capper D, Jones DTW, Sill M (2018). DNA methylation-based classification of central nervous system tumours. Nature [Internet].

[8] Adeberg S, Knoll M, Koelsche C (2022). DNA-methylome-assisted classification of patients with poor prognostic subventricular zone associated IDH-wildtype glioblastoma. Acta Neuropathol [Internet].

[9] Newell F, Pires da Silva I, Johansson PA (2022). Multiomic profiling of checkpoint inhibitor-treated melanoma: Identifying predictors of response and resistance, and markers of biological discordance. Cancer Cell [Internet].

[10] Duruisseaux M, Martínez-Cardús A, Calleja-Cervantes ME (2018). Epigenetic prediction of response to anti-PD-1 treatment in non-small-cell lung cancer: a multicentre, retrospective analysis. Lancet Respir Med [Internet].

[11] Bhan A, Soleimani M, Mandal SS (2017). Long Noncoding RNA and Cancer: A New Paradigm. Cancer Res [Internet].

[12] Zhong C, Tao B, Li X (2022). HOXA-AS2 contributes to regulatory T cell proliferation and immune tolerance in glioma through the miR-302a/KDM2A/JAG1 axis. Cell Death Dis [Internet].

[13] Wang Y, Yi K, Liu X (2021). HOTAIR Up-Regulation Activates NF-κB to Induce Immunoescape in Gliomas. Front Immunol [Internet].

[14] Wang Z, Yang B, Zhang M (2018). lncRNA Epigenetic Landscape Analysis Identifies EPIC1 as an Oncogenic lncRNA that Interacts with MYC and Promotes Cell-Cycle Progression in Cancer. Cancer Cell [Internet].

[15] Ceccarelli M, Barthel FP, Malta TM (2016). Molecular Profiling Reveals Biologically Discrete Subsets and Pathways of Progression in Diffuse Glioma. Cell [Internet].

[16] Roadmap Epigenomics Consortium, Kundaje A, Meuleman W (2015). Integrative analysis of 111 reference human epigenomes. Nature [Internet].

[17] Quinlan AR, Hall IM (2010). BEDTools: a flexible suite of utilities for comparing genomic features. Bioinformatics [Internet].

[18] Wilkerson MD, Hayes DN (2010). ConsensusClusterPlus: a class discovery tool with confidence assessments and item tracking. Bioinformatics [Internet].

[19] Li T, Fu J, Zeng Z (2020). TIMER2.0 for analysis of tumor-infiltrating immune cells. Nucleic Acids Res [Internet].

[20] Li Y, Xu S, Xu D (2022). Pediatric Pan-Central Nervous System Tumor Methylome Analyses Reveal Immune-Related LncRNAs. Front Immunol [Internet].

[21] Thorsson V, Gibbs DL, Brown SD (2018). The Immune Landscape of Cancer. Immunity [Internet].

[22] Barbie DA, Tamayo P, Boehm JS (2009). Systematic RNA interference reveals that oncogenic KRAS-driven cancers require TBK1. Nature [Internet].

[23] Yoshihara K, Shahmoradgoli M, Martínez E (2013). Inferring tumour purity and stromal and immune cell admixture from expression data. Nat Commun [Internet].

[24] Lauss M, Donia M, Harbst K (2017). Mutational and putative neoantigen load predict clinical benefit of adoptive T cell therapy in melanoma. Nat Commun [Internet].

[25] Rooney MS, Shukla SA, Wu CJ, Getz G, Hacohen N (2015). Molecular and genetic properties of tumors associated with local immune cytolytic activity. Cell [Internet].

[26] Jiang P, Gu S, Pan D (2018). Signatures of T cell dysfunction and exclusion predict cancer immunotherapy response. Nat Med [Internet].

[27] Mathewson ND, Ashenberg O, Tirosh I (2021). Inhibitory CD161 receptor identified in glioma-infiltrating T cells by single-cell analysis. Cell [Internet].

[28] Bhattacharya S, Dunn P, Thomas CG (2018). ImmPort, toward repurposing of open access immunological assay data for translational and clinical research. Sci data [Internet].

[29] Li Y, Jiang T, Zhou W (2020). Pan-cancer characterization of immune-related lncRNAs identifies potential oncogenic biomarkers. Nat Commun [Internet].

[30] Zhou Y, Zhou B, Pache L (2019). Metascape provides a biologist-oriented resource for the analysis of systems-level datasets. Nat Commun [Internet].

[31] Breeze CE, Reynolds AP, van Dongen J (2019). eFORGE v2.0: updated analysis of cell type-specific signal in epigenomic data. Bioinformatics [Internet].

[32] Grabovska Y, Mackay A, O'Hare P (2020). Pediatric pan-central nervous system tumor analysis of immune-cell infiltration identifies correlates of antitumor immunity. Nat Commun [Internet].

[33] Lu C, Klement JD, Ibrahim ML (2019). Type I interferon suppresses tumor growth through activating the STAT3-granzyme B pathway in tumor-infiltrating cytotoxic T lymphocytes. J Immunother cancer [Internet].

[34] Wu Y, Fletcher M, Gu Z (2020). Glioblastoma epigenome profiling identifies SOX10 as a master regulator of molecular tumour subtype. Nat Commun [Internet].

[35] Cao M, Wang L, Xu D (2022). The synergistic interaction landscape of chromatin regulators reveals their epigenetic regulation mechanisms across five cancer cell lines. Comput Struct Biotechnol J [Internet].

[36] Bhutani N, Burns DM, Blau HM (2011). DNA demethylation dynamics. Cell [Internet].

[37] Park E-G, Pyo S-J, Cui Y, Yoon S-H, Nam J-W (2022). Tumor immune microenvironment lncRNAs. Brief Bioinform [Internet].

[38] Jin K, Wang S, Zhang Y (2019). Long non-coding RNA PVT1 interacts with MYC and its downstream molecules to synergistically promote tumorigenesis. Cell Mol Life Sci [Internet].

[39] Casey SC, Tong L, Li Y (2016). MYC regulates the antitumor immune response through CD47 and PD-L1. Science [Internet].

[40] Pearson JRD, Cuzzubbo S, McArthur S (2020). Immune Escape in Glioblastoma Multiforme and the Adaptation of Immunotherapies for Treatment. Front Immunol [Internet].

[41] Gangoso E, Southgate B, Bradley L (2021). Glioblastomas acquire myeloid-affiliated transcriptional programs via epigenetic immunoediting to elicit immune evasion. Cell [Internet].

[42] Xu D, Wang L, Pang S (2021). The Functional Characterization of Epigenetically Related lncRNAs Involved in Dysregulated CeRNA-CeRNA Networks Across Eight Cancer Types. Front cell Dev Biol [Internet].

[43] Dhatchinamoorthy K, Colbert JD, Rock KL (2021). Cancer Immune Evasion Through Loss of MHC Class I Antigen Presentation. Front Immunol [Internet].

[44] Wang S, Wang R, Gao F, Huang J, Zhao X, Li D (2022). Pan-cancer analysis of the DNA methylation patterns of long non-coding RNA. Genomics [Internet].

[45] Li G, Kryczek I, Nam J (2021). LIMIT is an immunogenic lncRNA in cancer immunity and immunotherapy. Nat Cell Biol [Internet].

[46] Zhou M, Zhang Z, Bao S (2021). Computational recognition of lncRNA signature of tumor-infiltrating B lymphocytes with potential implications in prognosis and immunotherapy of bladder cancer. Brief Bioinform [Internet].

[47] Zheng Y, Tian X, Wang T (2019). Long noncoding RNA Pvt1 regulates the immunosuppression activity of granulocytic myeloid-derived suppressor cells in tumor-bearing mice. Mol Cancer [Internet].

[48] Gieryng A, Pszczolkowska D, Walentynowicz KA, Rajan WD, Kaminska B (2017). Immune microenvironment of gliomas. Lab Invest [Internet].

[49] Balasubramaniyan V, Bhat KP (2017). Targeting MIR155HG in glioma: a novel approach. Neuro Oncol [Internet].

[50] Simonds EF, Lu ED, Badillo O (2021). Deep immune profiling reveals targetable mechanisms of immune evasion in immune checkpoint inhibitor-refractory glioblastoma. J Immunother cancer [Internet].

[51] Carosella ED, Rouas-Freiss N, Tronik-Le Roux D, Moreau P, LeMaoult J (2015). HLA-G: An Immune Checkpoint Molecule. Adv Immunol [Internet].

