# Supplementary material for: Epigenetically regulated lncRNAs dissect the intratumoural heterogeneity and facilitate immune evasion of glioblastomas
Source: Theranostics. 2023 Mar 5;13(5):1490–505. doi: 10.7150/thno.79874 (PMC10086206; doi:10.7150/thno.79874)
Supplement: Supplementary file 1 — Supplementary figures. [file thnov13p1490s1.pdf]

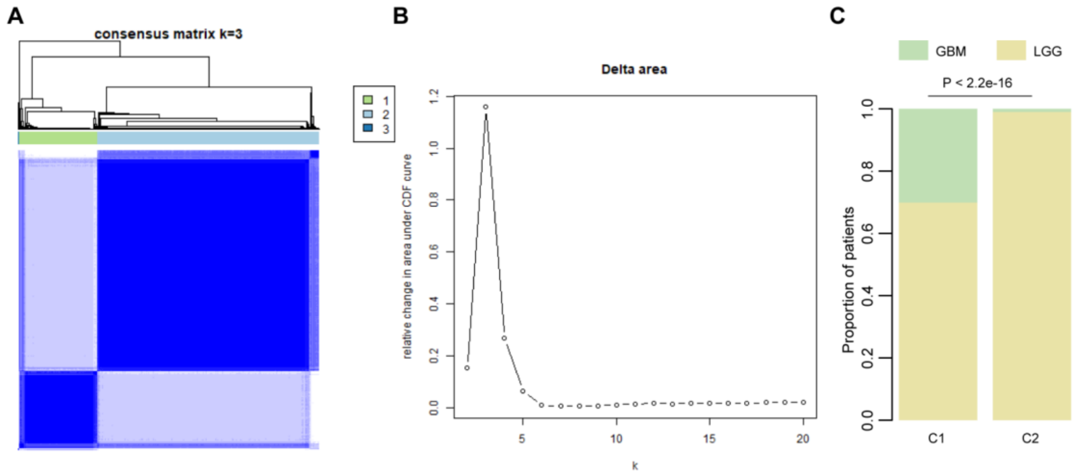

Figure S1. LncRNA methylation defines stable subtypes for glioma patients. (A) Consensus clustering matrix of glioma cancer samples for k = 3. (B) Relative change in area under CDF curve for k = 2 to k = 20. (C) The proportions of LGG and GBM samples in C1 and C2 subtypes.

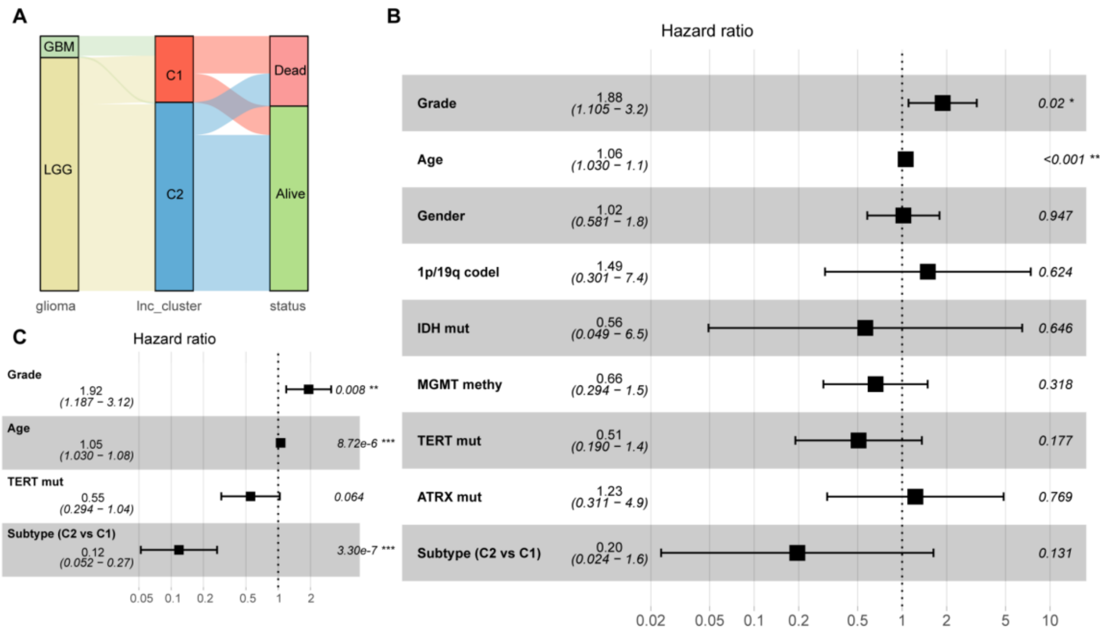

Figure S2. The clinical outcomes of glioma patients and prognostic independence evaluation. (A) The clinical outcomes of glioma subtypes based on lncRNA methylation features. (B) Forest plot for the multiple Cox proportional hazards model of glioma. (C) Forest plot for the stepwise Cox proportional hazards model of glioma.

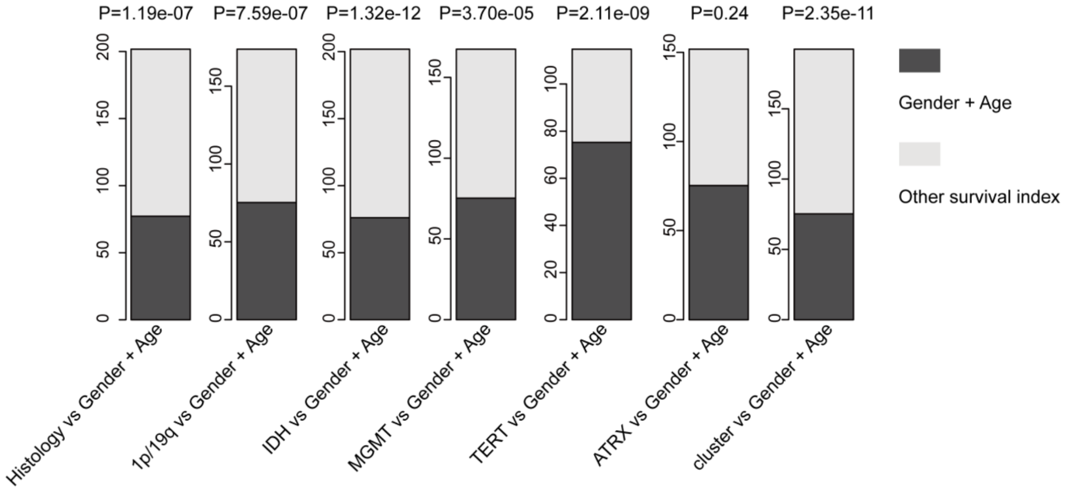

Figure S3. The estimated log-likelihood ratio statistic of a Cox proportional hazards model. Original clusters represent the Cox model constructed by gender and age. Additional clinical information was included in subsequent Cox risk models, respectively. The change of LR statistic as features were added to the model was assessed for significance by Chi-square tests.

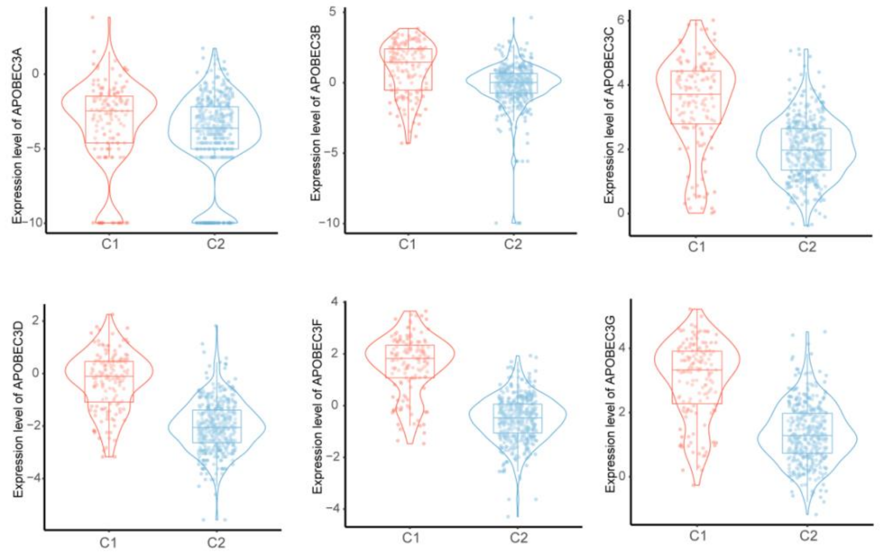

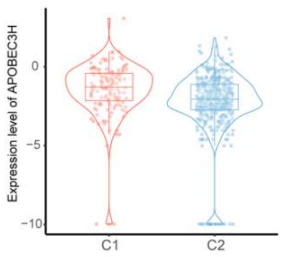

	LogFC	LogCPM	LR	Pvalue	FDR
APOBEC3A	0.9832	-2.2361	31.8293	1.68E-08	3.84E-08
APOBEC3B	1.3010	0.9127	129.7255	4.71E-30	3.23E-29
APOBEC3C	1.5584	3.9556	298.3113	7.69E-67	2.04E-65
APOBEC3D	1.6048	-0.5673	333.2857	1.85E-74	6.37E-73
APOBEC3F	1.8790	0.5884	621.8392	2.98E-137	4.90E-135
APOBEC3G	1.6488	2.5634	383.9306	1.73E-85	8.25E-84
APOBEC3H	0.7661	-2.0451	39.7918	2.83E-10	7.22E-10

Figure S4. Expression patterns of APOBEC3 gene family between glioma subtypes.

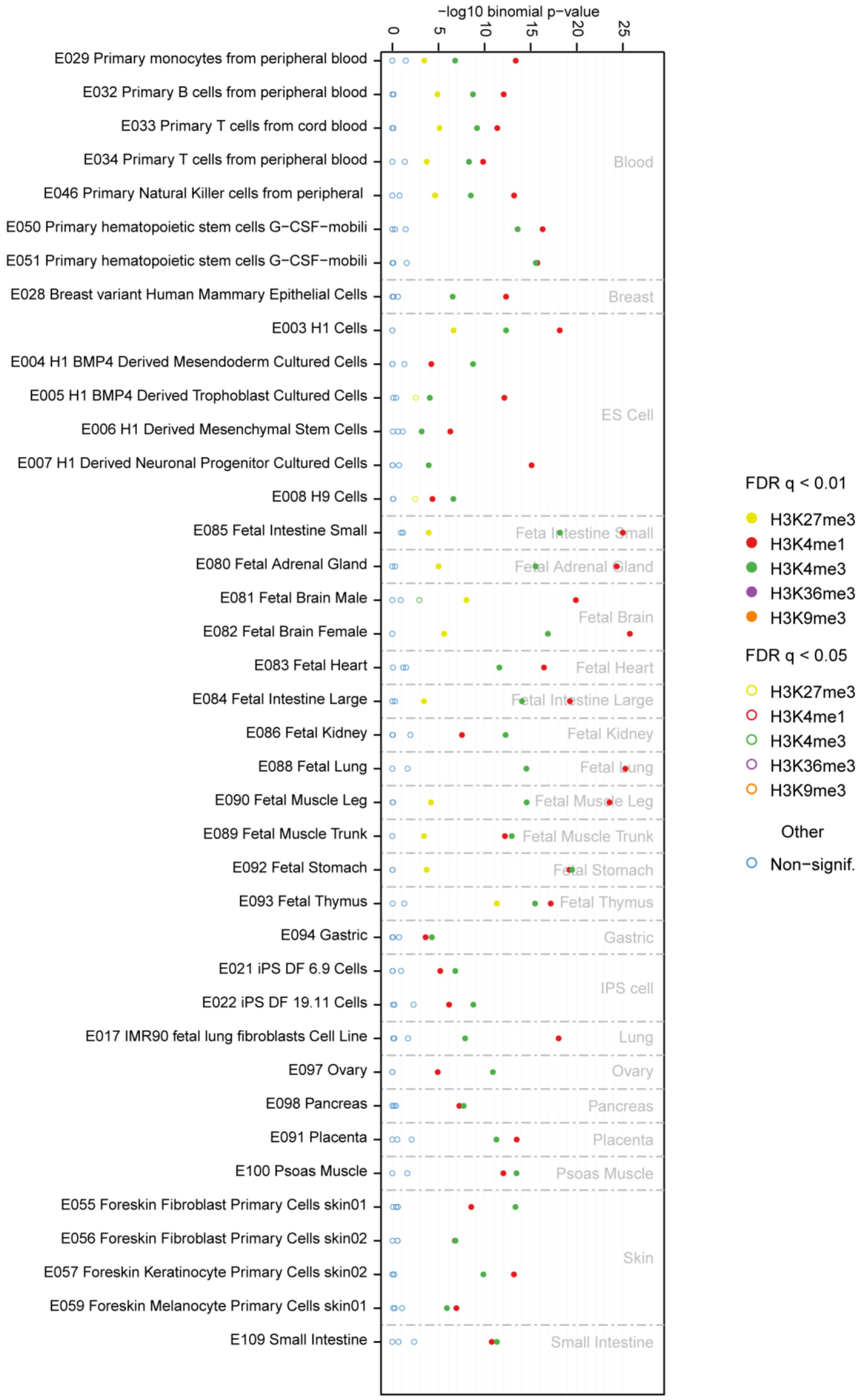

Figure S5. The enrichment between CpG probes belonging to the promoter of ER lncRNAs and histone modification markers via eForge tool.

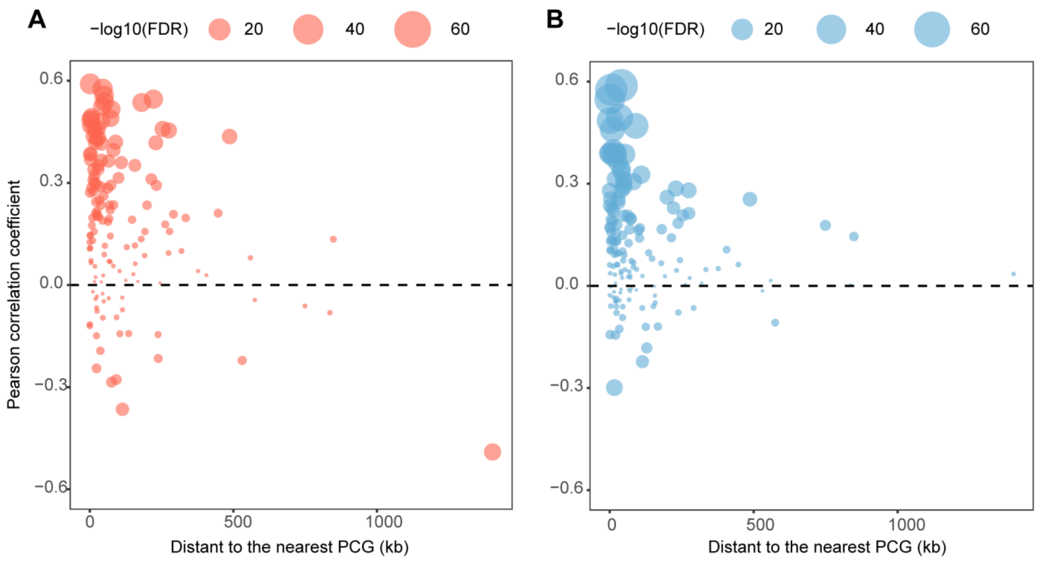

Figure S6. Relationships between ER lncRNAs expression and nearest PCG expression in (A) C1 cluster and (B) C2 cluster.

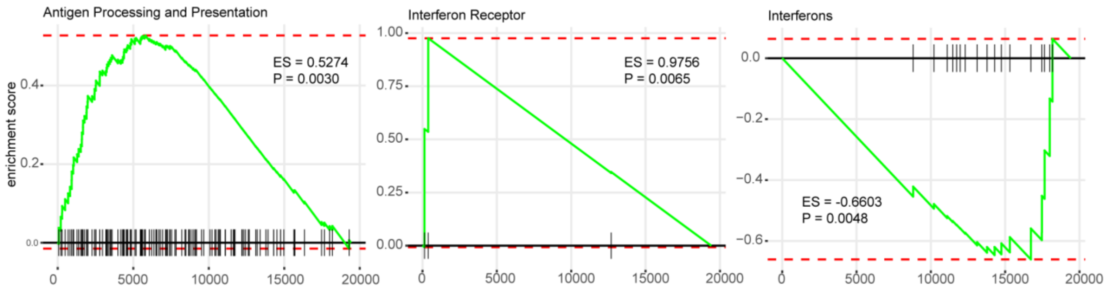

Figure S7. The correlated genes for lncRNA PVT1 were significantly enriched in antigen processing and presentation, interferon receptor and interferons in C1 cluster.

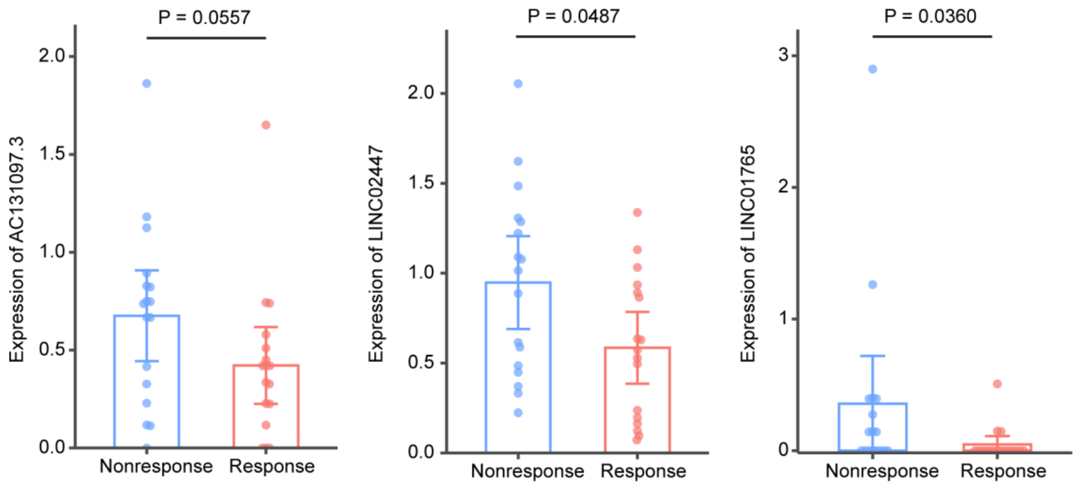

Figure S8. Expression level of lncRNA AC131097.3, LINC02447 and LINC01765 in response and nonresponse glioma patients received anti-PD1 immunotherapy.

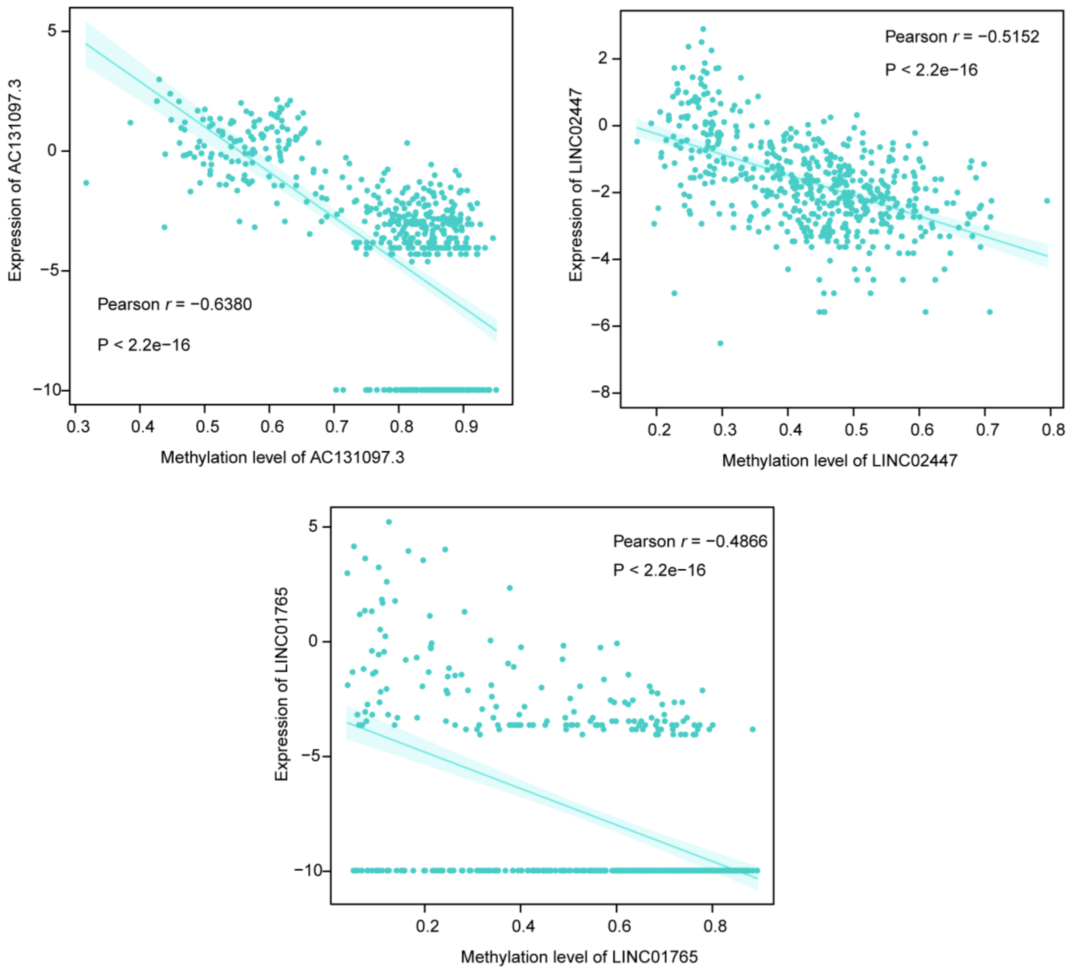

Figure S9. Correlation between the expression and methylation level of AC131097.3, LINC02447 and LINC01765 in TCGA glioma cohort.

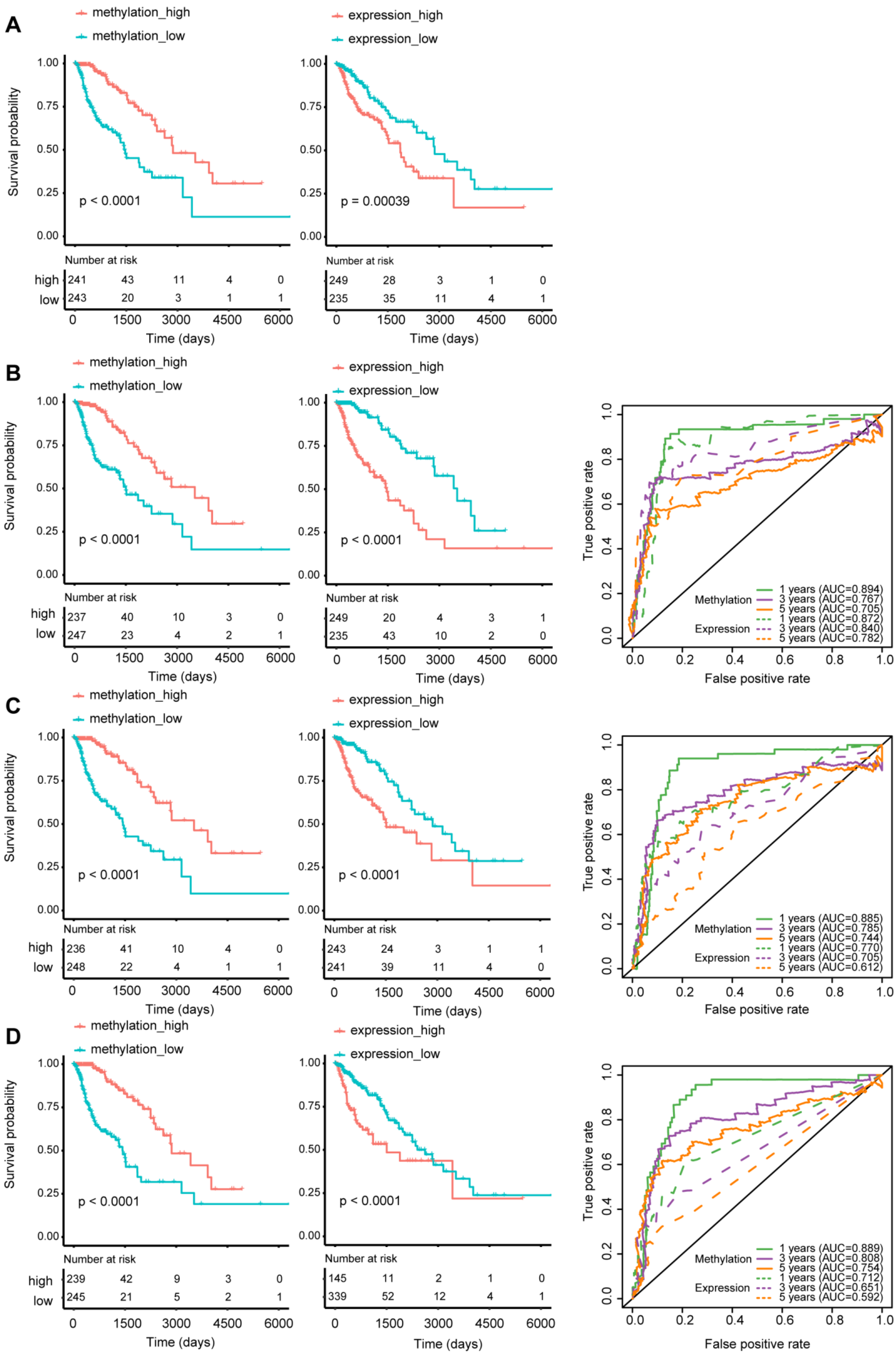

Figure S10. Prognosis efficacy of methylation and expression of ER lncRNAs for glioma patients in TCGA cohort. (A) CD109-AS1, (B) AC131097.3, (C) LINC02447 and (D) LINC01765. Left panel is the Kaplan–Meier plot of glioma patients based on the median methylation level of ER

lncRNAs. Midddle panel is the Kaplan–Meier plot of glioma patients based on the median expression level of ER lncRNAs. Right panel is the comparison of prognosis efficacy between methylation and expression of ER lncRNAs.

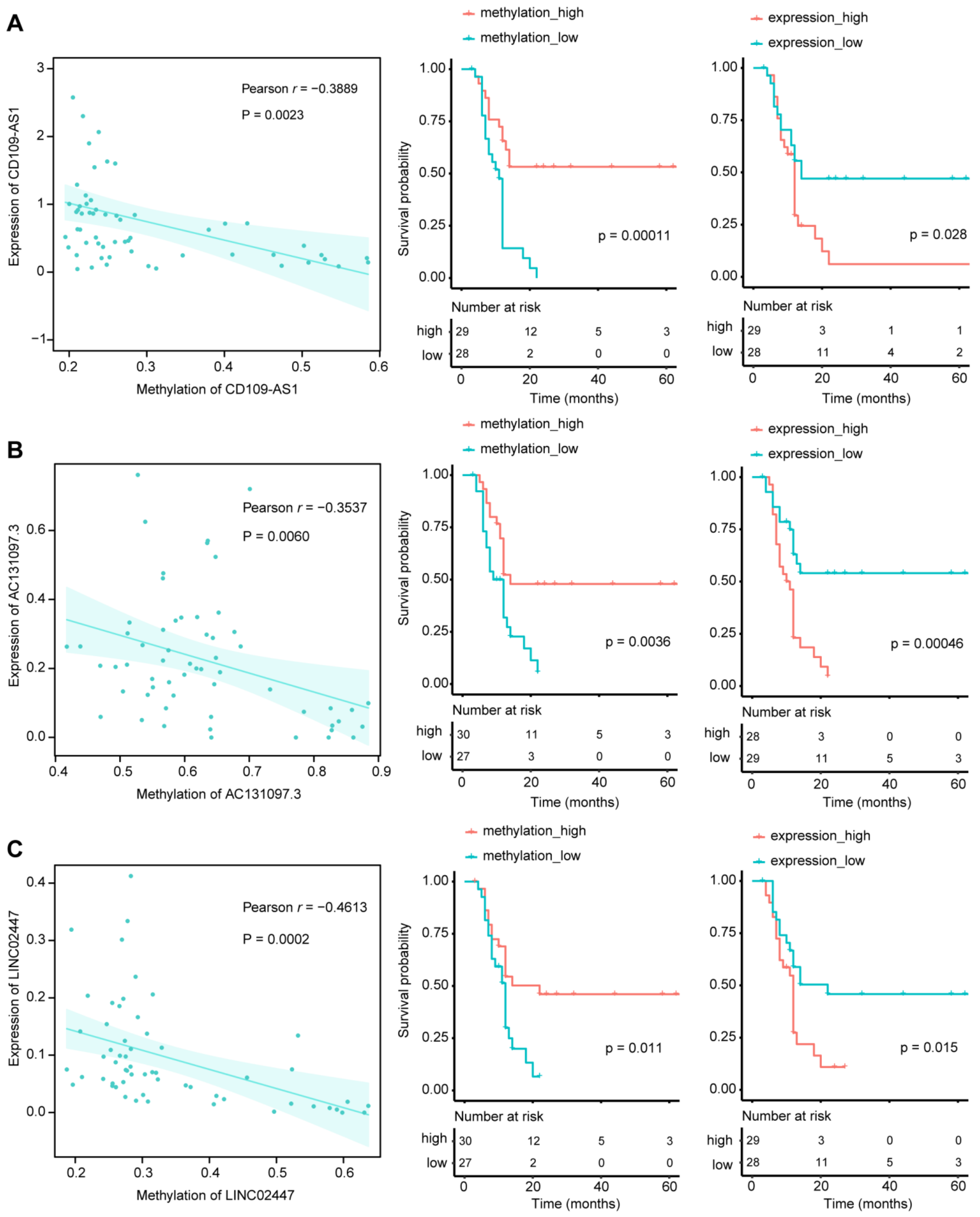

Figure S11. The validation of ER lncRNAs in external dataset. (A) CD109-AS1, (B) AC131097.3 and (C) LINC02447. Left panel is the correlation between methylation and expression level of ER lncRNAs. Middle panel is the Kaplan–Meier plot of glioma patients based on the median methylation level of ER lncRNAs. Right panel is the Kaplan–Meier plot of glioma patients based on the median expression level of ER lncRNAs.

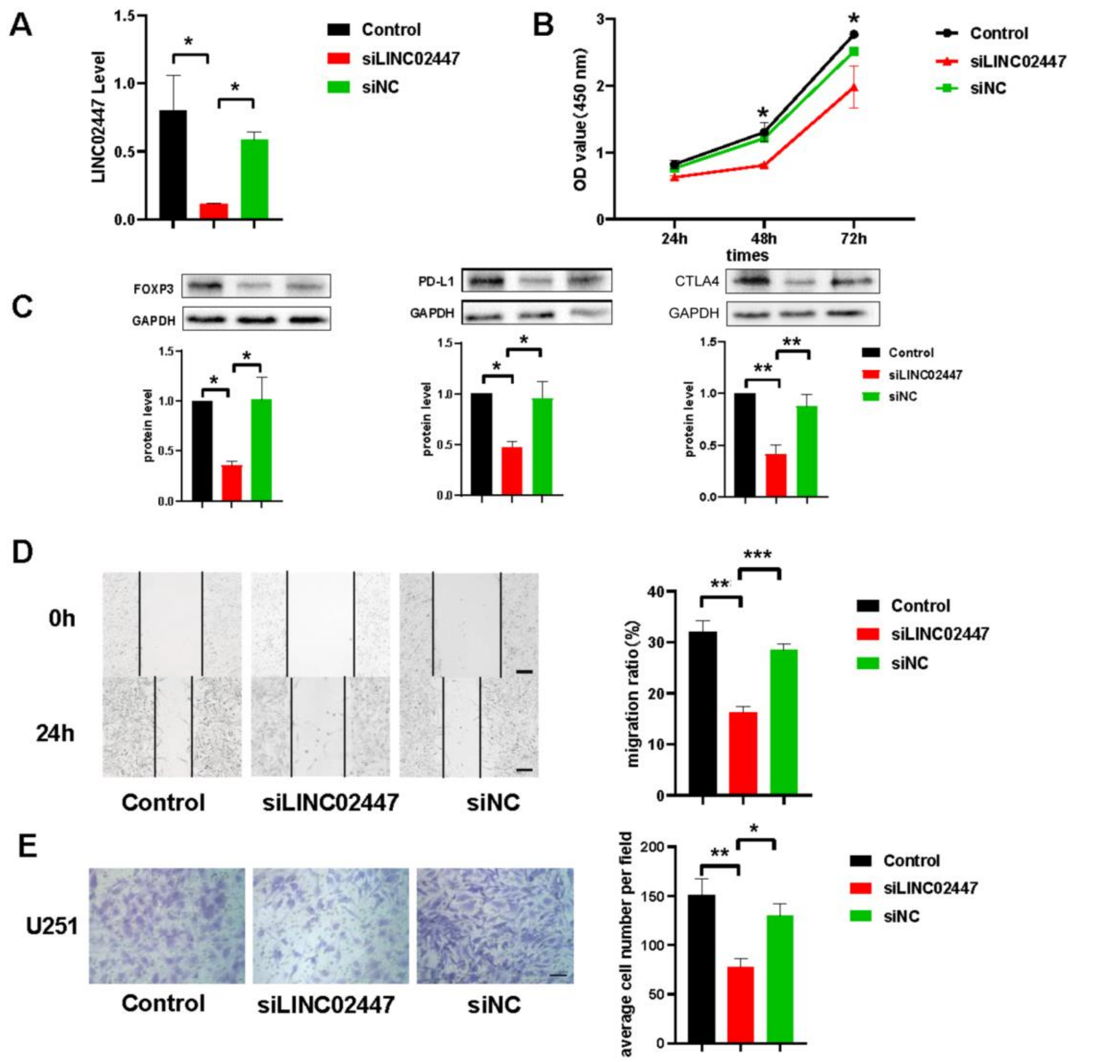

Figure S12. Role of LINC02447 in the proliferation, migration, invasion and immune evasion of GBM cell lines. (A) The expression of LINC02447 in U251 cells transduced with siRNA was examined by qRT-PCR (n = 3-5, **p < 0.01). (B), CCK-8 assays show that the inhibition of LINC02447 decreased cell proliferation in U251 cell lines cells. (n = 6, *p < 0.05, **p < 0.01). (C) The expression of FOXP3, CTLA-4 and PD-L1 in U251 cells transduced with LINC02447 siRNA was examined by Western blot; GAPDH was an internal control (n = 3, *p < 0.05, **p < 0.01). (D) Wound healing assays show that LINC02447 knockdown significantly reduced the cell migration ability of U251 cells with the representative images on the left and the quantitative analysis on the right (n = 3, ***p < 0.001). (E) Transwell invasion of LINC02447 siRNA GBM cells is significantly reduced compared with control cells (n = 3, **p < 0.01).

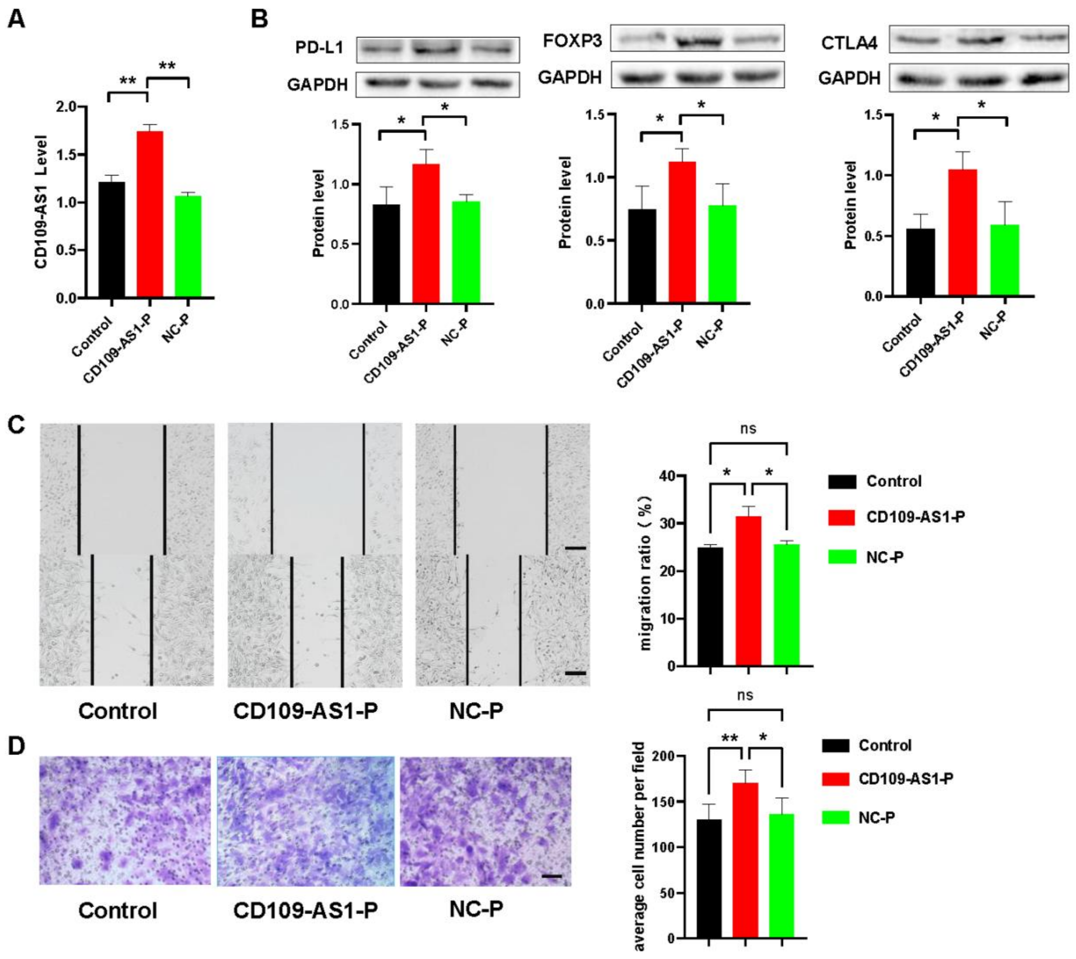

Figure S13. CD109-AS1 drives the migration and invasion of GBM cell lines. (A) The expression of CD109-AS1 in U251 cells was determined by qRT-PCR. (n = 3, **p < 0.01). (B) The expression of FOXP3, CTLA-4 and PD-L1 in U251 cells transduced with CD109-AS1-P was examined by Western blot; GAPDH was an internal control (n = 3, *p < 0.05). (C) Wound healing assays show that CD109-AS1 enhanced the cell migration ability of U251 cells with the representative images on the left and the quantitative analysis on the right (n = 3, *p < 0.05). (D) Transwell experiment showed that CD109-AS1 overexpression increased cell invasion compared with control cells(n = 3, *p < 0.05，**p < 0.01)

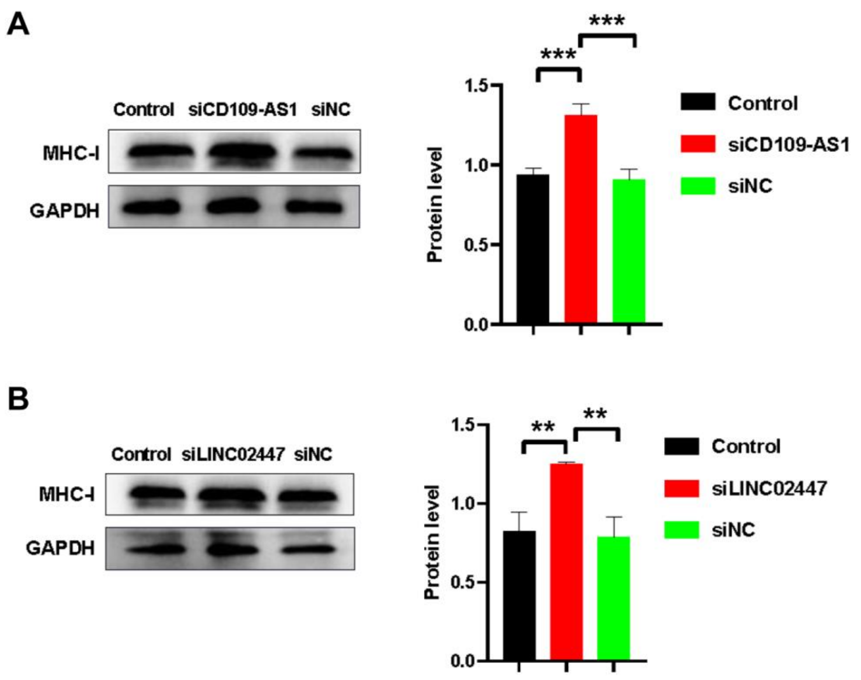

Figure S14. The expression of MHC-I in U251 cells. (A) Transduced with CD109-AS1 and (B) Transduced with LINC02447 siRNA was examined by Western blot. GAPDH was an internal control (n = 3, *p < 0.05, **p < 0.01).

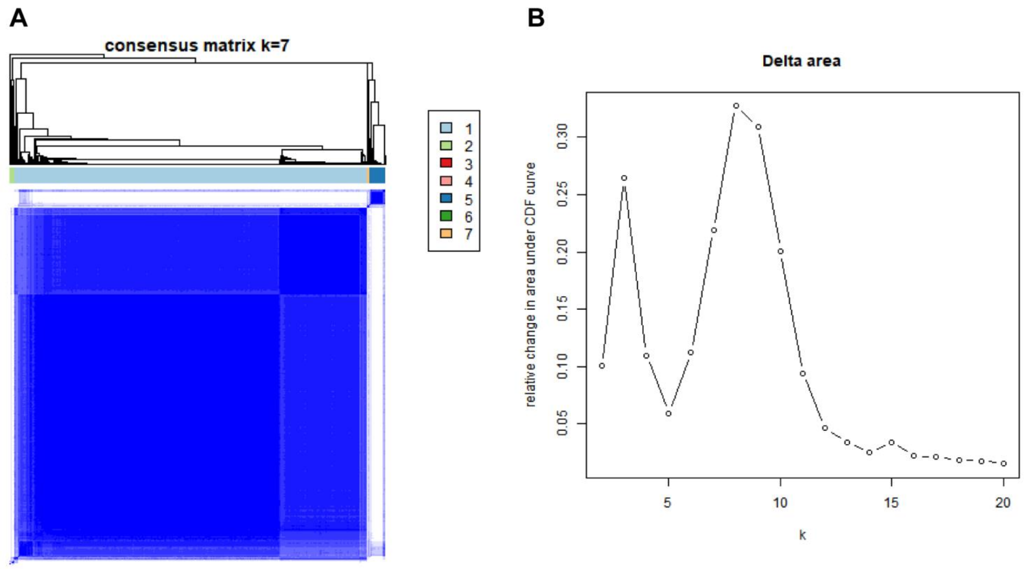

Figure S15. The glioma subtypes defined by mRNA methylation. (A) Consensus clustering matrix of glioma cancer samples for k = 7. (B) Relative change in area under CDF curve for k = 2 to k = 20.

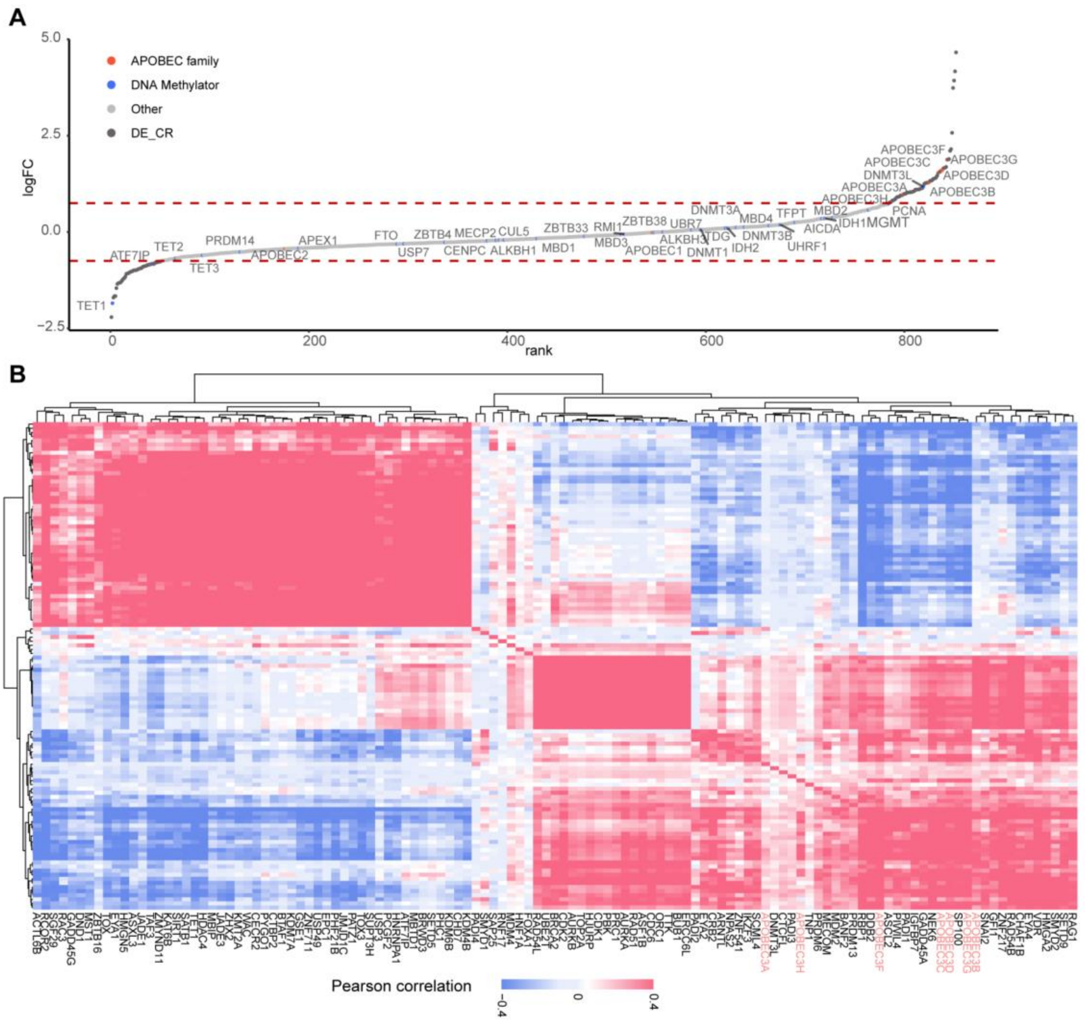

Figure S16. The expression pattern of APOBEC3 family in glioma patients. (A) The deviation of expression levels for CRs in glioma subtypes. CRs were ranked based on the fold change of C1 compared to C2. (B) The identification of the co-regulated modules for differently expressed CRs.

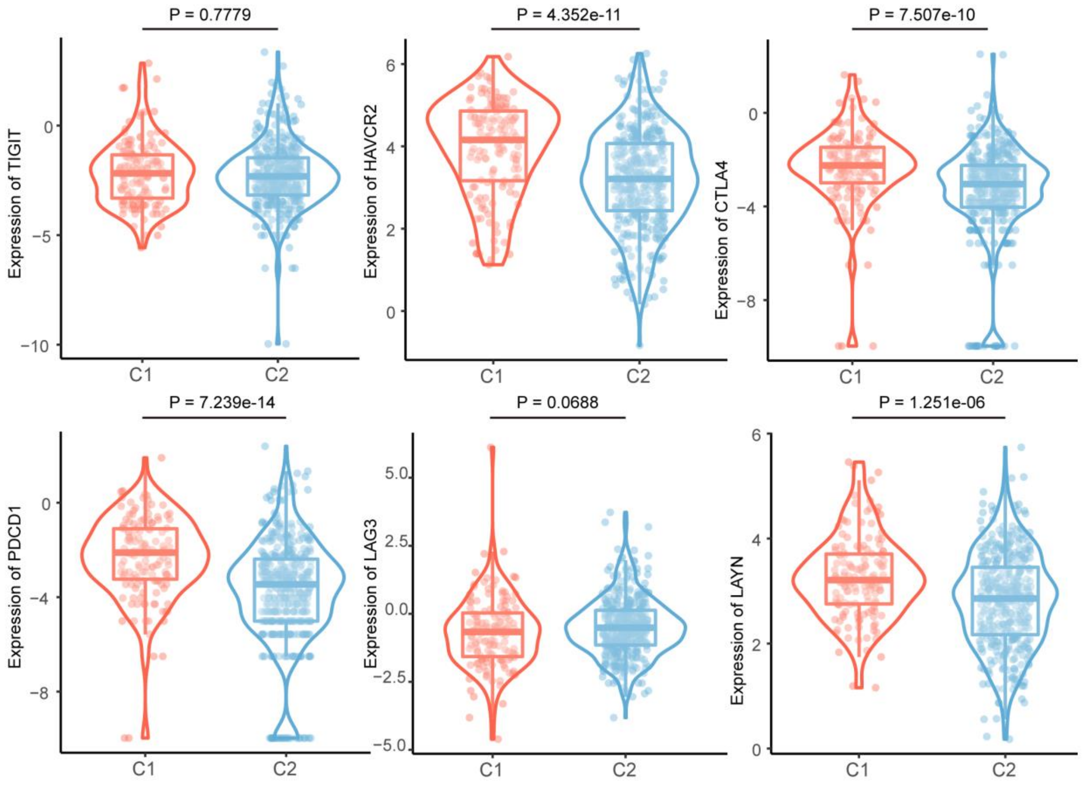

Figure S17. The expression pattern of T cell exhaustion markers in glioma patients.
